# Dynamic heterogeneity in the self-induced spin glass state of elemental neodymium

**DOI:** 10.1038/s41467-026-74637-1

**Published:** 2026-06-20

**Authors:** Lorena Niggli, Julian H. Strik, Karoline Oetker, Zhengyuan Liu, Anders Bergman, Nadine Hauptmann, Mikhail I. Katsnelson, Daniel Wegner, Alexander A. Khajetoorians

**Affiliations:** 1 Institute for Molecules and Materials, Radboud University, Nijmegen, The Netherlands; 2 Department of Physics and Astronomy, Uppsala University, Uppsala, Sweden; 3 Wallenberg Initiative Materials Science for Sustainability, Uppsala University, Uppsala, Sweden

**Keywords:** Condensed-matter physics, Magnetic properties and materials

## Abstract

Spin glasses are magnetic materials exhibiting numerous magnetization patterns, that randomly vary both in real space and in time. To date, it is still not well understood what the nature of these spatiotemporal dynamics is, namely if they are completely random or if there are relevant and correlated length and time scales. Here, we demonstrate dynamic heterogeneity in the aging dynamics of elemental neodymium. We used spin-polarized scanning tunneling microscopy in combination with atomistic spin dynamics simulations to image the locally ordered magnetic patterns in the self-induced spin glass state and tracked the induced spatiotemporal dynamics in response to external perturbations. We observed that the real-space magnetization exhibited a coexistence of slow and fast dynamics coinciding with particular length scales. We adapted a wavelet paradigm to identify and correlate local spatial order with its dynamical evolution. These results provide a platform to study dynamics in spin glasses beyond the mean-field limit, linking to a generalized picture of glasses.

## Abstract (web summary)

Dynamical heterogeneity relates to particular local length scales in glassy materials that persist over time. While well studied across a range of glassy materials, its presence in spin glasses remains unclear due to the requirement of nanoscale imaging. Here, using spin-polarized scanning tunnelling microscopy, Niggli et al image dynamic heterogeneity in real space in a self-induced spin glass.

## Introduction

The complexity of spin glasses continues to be the subject of intense experimental and theoretical interest. Its magnetic state is often described as a distribution of seemingly random patterns that continuously evolve^1,2^, driven by the combination of competing exchange interactions and disorder. Dynamics in spin glasses, often referred to as aging, are characterized by the coexistence of multiple relaxation times spanning different orders of magnitude, where the magnetic state at a given time depends on its history, or age^2,3^. Aging dynamics distinguishes spin glasses from other frustrated magnets due to their non-ergodic behavior^1,3^. Demonstrating non-ergodicity theoretically was most convincingly achieved^4–7^ using replica symmetry breaking (RSB) to solve the Sherrington–Kirkpatrick model^8^, where magnetic coupling extends to infinite range (i.e., infinite dimension). The RSB approach yields a solution that is length invariant, i.e., there are no favorable length scales that define particular domains composed of distinct patterns. Furthermore, it is solvable only in the limit of infinite-radius interactions, which is common for theories based on a mean-field approach. In contrast, there is a long-standing debate about the applicability of this mean-field picture to describe spin glass materials^9–11^, including the role of dimensionality and short-range interactions. To this end, there is extensive theoretical work based on the Edwards–Anderson model^12,13^, in which the exchange interaction is truncated at a finite range, where concepts like droplet scaling^14–17^ have been developed to address the role of short-range phenomena in finite dimensions. For short-range models, there is still no completely rigorous consideration that captures ergodicity breaking^9^, resulting in an open theoretical question of how to describe aging dynamics in the presence of local order.

Experiments performed on spin glasses often show both aging behavior as well as the emergence of short-range order defined by a local length scale^18–21^, necessitating theoretical descriptions that go beyond the mean-field description. It was shown that memory and rejuvenation can be retained during different aging cycles^22,23^, which has been described by considering hierarchies of metastable states with different energetic favorability^24–26^. Nevertheless, experiments that can provide insight into the interplay between local length scales and aging dynamics are often challenging^1^: it is usually difficult to disentangle the role of disorder and the emergence of short-range order (e.g., due to defects)^2^, and coinciding aging dynamics with the role of short-range order requires experimental techniques that can combine spatial with temporal resolution. To this end, the self-induced spin glass^27–29^, in which glassy behavior emerges in the absence of disorder^30^, provides a materials platform to study aging dynamics beyond the conventional approximations.

Dynamic heterogeneity (DH) is a phenomenon in glassy materials describing the emergence of local length scales that persist in time, which are correlated with a subset of available time scales. DH goes beyond the mean-field description, but to date has been poorly studied experimentally in spin glasses, due to the aforementioned challenges related to disorder and local imaging. Additionally, there are a limited number of theoretical investigations describing DH and its effects on the dynamics near the spin glass transition^31–34^. In contrast to spin glasses, DH is an ubiquitous phenomenon observed in amorphous solids^35^. DH in amorphous materials has provided a means to study glassy dynamics beyond the mean-field limit, and various methods have been developed to quantify the correlations between length and time scales^36–40^. In such materials, nucleation behavior has been studied, where regions in real space show a structured dynamical evolution^41–45^. The observation and study of DH in spin glasses provides a means to investigate glassy behavior in magnetic materials beyond the mean field limit and also provides a natural bridge toward unifying the concepts of spin glasses and structural glasses.

## Results and discussion

### Manifestation of dynamic heterogeneity and the self-induced spin glass in neodymium

**Fig. 1:**
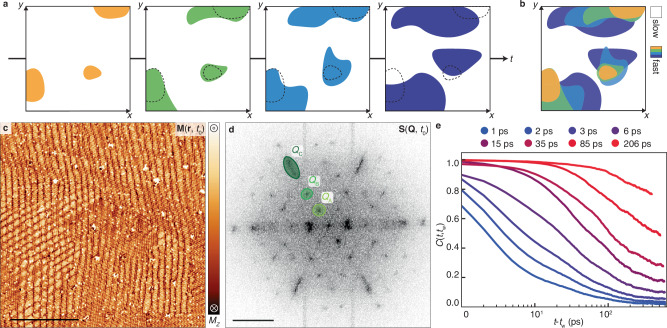
Dynamic heterogeneity in the evolution of **M**(**r**, *t*) and the self-induced spin glass state of elemental neodymium. **a** Four sketches of the spatially resolved magnetization **M**(**r**, *t*) at subsequent times *t*. Each **M**(**r,** *t*) shows a coexistence of relatively slower (white) and faster (colored regions) dynamics. The dashed lines illustrate the initial extent of the faster regions in (**a**) to demonstrate their spatial correlation over time. Unlike a random evolution, in which the different dynamics show no link to specific regions in real space, here, there are correlations between length and time scales reminiscent of DH. **b** Compilation of all the sketches in (**a**) to illustrate persistence behavior that is linked to spatiotemporal correlations resulting from dynamic heterogeneity. **c** Magnetization image **M**(**r**, *t*_0_) measured at *T* = 1.3 K and *B* = 0 T after zero-field cooling exhibits spatially varying multi-*Q* order. **d** Static magnetic structure factor **S**(**Q**, *t*_0_) obtained by fast Fourier transformation of (**c**) is characterized by three *Q*-pockets along each high-symmetry axis, labeled *Q*_A_, *Q*_B_, and *Q*_C_ (image parameters: *I*_t_ = 200 pA; scale bar **M**(**r**, *t*_0_): 50 nm, scale bar **S**(**Q,** *t*_0_): 3 nm^−1^, color scale for **S**(**Q**)-image is a linear gradient; defect density: 0.0045 ML). **e** Simulated autocorrelation function *C*(*t*, *t*_w_) for different waiting times *t*_w_. A glassy relaxation behavior is seen even though the waiting times *t*_w_ span three orders of magnitude.

We illustrate a schematic of how DH can be studied by imaging the local magnetization in real space, **M**(**r**, *t*), at different instances in time *t* (Fig. 1a). Comparing two initial consecutive snapshots in time, there are subsets of the total area that exhibit a dynamical evolution from the previous state (illustrated by the colored regions). DH manifests in real space as further instances are sampled: the spatial location of the evolving regions are not randomized over time, but rather the initial regions that show a temporal evolution change in shape, i.e., they grow from the initially nucleated areas. A compilation of these sequences is shown schematically in Fig. 1b. This behavior, where there are spatiotemporal correlations, is in contrast to a mean-field picture, where **M**(**r**, *t*) should randomize as there are no favorable length scales persisting in time.

Here, we quantified DH in the self-induced spin glass state of elemental neodymium^46^ by studying the evolution of the spatially resolved magnetization at different instances in time using spin-polarized scanning tunneling microscopy (SP-STM). The self-induced spin glass state of Nd(0001) is characterized by the coexistence of multiple and distinct locally ordered magnetic patterns observable in the real-space surface magnetization **M**(**r**, *t*), as seen in Fig. 1c (see “Methods” and Supplementary Fig. S1) and previously detailed in refs. ^46,47^. Each local pattern had no well-defined domain boundary and could therefore not easily be delineated from other neighboring patterns. The fast Fourier transform (FFT) of the real-space magnetization **M**(**r**, *t*), which we refer to as **S**(**Q**, *t*), is closely related to the static magnetic structure factor^46^. **S**(**Q**, *t*) in Fig. 1d exhibited three distinct distributions of degenerate *Q*-states along each high-symmetry direction, which we refer to as *Q*_A_-, *Q*_B_- and *Q*_C_-pockets, respectively (radially outwards). |**Q**| = 2*π*⁄*λ* denotes a magnetic wave vector (*λ* is the real-space periodicity). The self-induced spin glass state is driven by frustrated interactions. Below a critical defect density, it is robust against the presence of weak disorder or a sparse number of defects^46^. This robustness stems from the nature of the long-range exchange interactions and the presence of many low-energy states separated by small energies. In this limit, defects can lead to small modulations of the interaction landscape and may modify the favorability of the various low-energy states^48^. The role of various defects on the self-induced spin glass state has been previously studied in ref. ^46^, and its effects on DH will be detailed below. Before discussing DH, we first checked, as in ref. ^46^, the simulated two-time autocorrelation function $C\left(t,{t}_{w}\right)$ to demonstrate aging dynamics in the ensemble used for simulation (see “Methods” for details), where $C\left(t,{t}_{w}\right)=\left\langle {{{\bf{m}}}}_{i}\left({t}_{w}\right){\cdot {{\bf{m}}}}_{i}(t+{t}_{w})\right\rangle$ with ${{{\bf{m}}}}_{i}$ denoting the magnetization of site *i, t* being time, and ${t}_{w}$ denoting the waiting time. The result is shown in Fig. 1e, which demonstrates the hallmark behavior of aging.

Aging in a spin glass is typically defined by the presence of multiple relaxation time scales, where the state of the ensemble depends on its history, i.e., its trajectory through phase space. In the traditional mean-field description of a spin glass^1–3^, the energy landscape is rugged and is characterized by a large number of local minima separated by energy barriers with a varying range of heights. This leads to a very broad distribution of typical time scales in the problem, which depend exponentially on the barrier heights due to thermal activation at any finite temperature. In the case of a self-induced spin glass, it is not clear if this mean-field picture, assuming infinitely long-range interactions, is a good approximation, as its energy landscape is defined by the given exchange frustration and can lead to a nontrivial spin structure factor. It was previously suggested that the self-induced spin glass can be related to the Hopfield network, in the limit where the return probability is negligible^48^. In this way, the self-induced spin glass and the structure of its energy landscape may strongly affect aging dynamics. Aging in elemental neodymium has previously been demonstrated through (a) calculations of the two-time correlation function revealing broken time translation invariance (see Fig. 1e and refs. ^46,47^) and experimentally by (b) evidence of multiple relaxation times below the glass transition temperature^46^. SP-STM-based methods provide the necessary spatial resolution to quantify the relevant length scales, but its time resolution is extremely limited. Therefore, to further quantify aging dynamics and study DH, it is necessary to show that sequential imaging with SP-STM, which reveals changes in the local magnetization, provides evidence of the history of the ensemble.

### Magnetic field cycling to anneal the self-induced spin glass

**Fig. 2:**
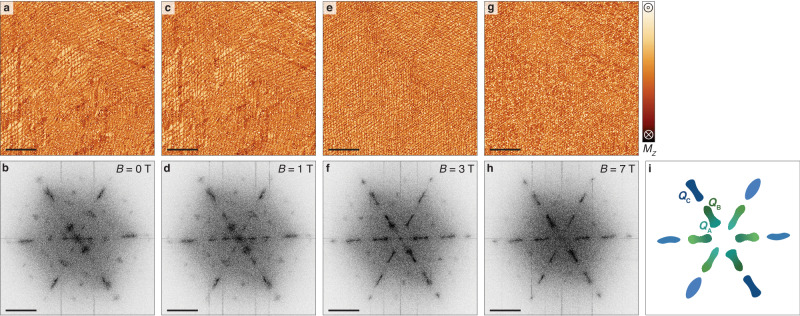
Magnetic field evolution of the self-induced spin glass state. Magnetization **M**(**r**) and the corresponding **S**(**Q**) images of the same area measured at *T* = 1.3 K in varying magnetic fields: **a**, **b** *B* = 0 T, **c**, **d** *B* = 1 T, **e**, **f** *B* = 3 T and **g**, **h** *B* = 7 T. Increasing the magnetic field does not favor a specific set of *Q*-states; instead, the spectral weight in the various *Q*-pockets broadens, and the patterns in **M**(**r**) become more disordered. **i** Sketch of the modified *Q*-pockets for *B* ≳2 T, where the *Q*_A_- and *Q*_B_-pockets shift towards each other and are linked by spectral weight appearing between them (image parameters: *I*_t_ = 200 pA; scale bar **M**(**r**): 50 nm, scale bar **S**(**Q**): 3 nm^−1^, color scale for **S**(**Q**)-images is a linear gradient).

**Fig. 3:**
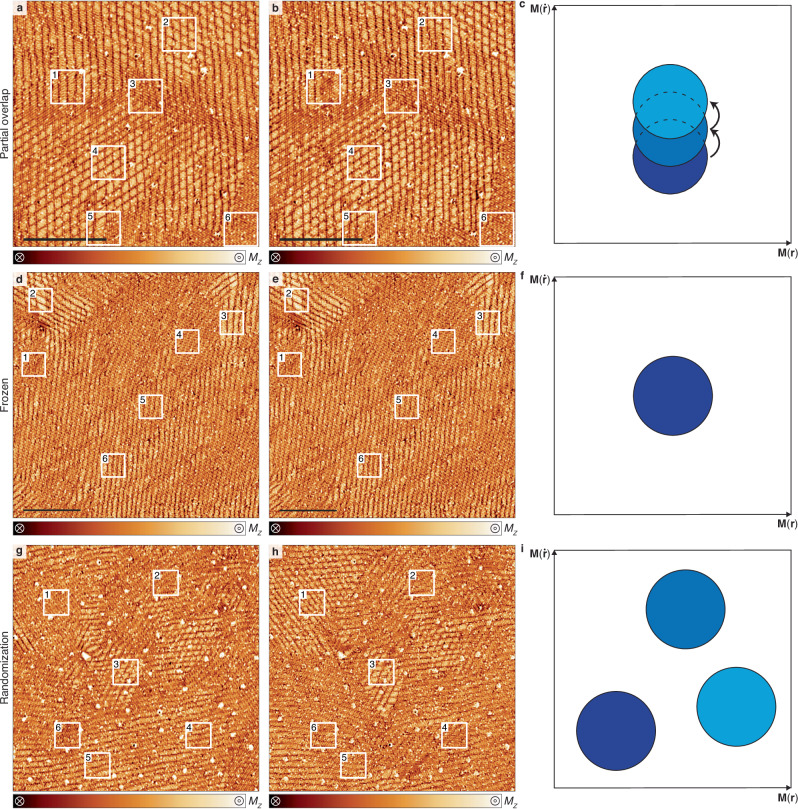
Imaging the evolution of the self-induced spin glass state after field-induced perturbations and phase space evolution. Two magnetization images measured at *T* = 1.3 K and *B* = 0 T of the same area after **a**, **b** applying a magnetic field cycle to *B* = 2 T showing partial overlap between the local multi-*Q* orders, **d**, **e** consecutively imaging the area without applying a perturbation leading to frozen magnetic patterns and **g**, **h** repeatedly heating and cooling across the glass transition temperature (*T*_g_ ~ 8 K) resulting in next to no correlations in the local order before and after re-entering the self-induced spin glass phase. These images were taken hours apart. The local areas outlined by white boxes are displayed in Fig. S3. **c**,**f**,**i** Sketch of a phase space picture for the three cases, where the ensemble of spins in a measured area is represented by a circle: **c** sizable correlations between subsequent images obtained by magnetic field cycling indicate partial overlap in phase space. **f** Without applying a perturbation, the system remains frozen, resulting in full overlap. **i** Upon re-entering the glassy phase by thermal cycling, the history is lost (image parameters: *I*_t_ = 200 pA; scale bar: 50 nm; **a**, **b** sample with defect density of 0.0045 ML, **d**, **e** sample with defect density of 0.0034 ML and **g**,**h** sample with defect density of 0.0068 ML).

In a self-induced spin glass, the ruggedness of the energy landscape can lead to the favorability of certain spin configurations. This affects its aging dynamics as well as defines a natural temperature, where the dynamics associated with specific configurations can be frozen out. To link the time-dependent behavior with local variations of the magnetization in real space, we first cooled the sample far below the glass transition temperature *T*_g_ = 8.1 K down to *T* = 1.3 K. At *T* = 1.3 K, the dynamics were effectively frozen out, thereby inhibiting any aging (Supplementary Fig. S2). In order to induce dynamics, we could not use elevated temperature, as entropy in this system is typically reduced with increasing temperature, below *T*_g_. This is due to the presence of a phase transition to an ordered multi-*Q* state above *T*_g_ = 8.1 K, as detailed in ref. ^47^. In this way, perturbations induced by temperature are not necessarily random, but strongly biased in phase space. In contrast, in ref. ^46^ and as shown in Fig. 2, we found that the application of an out-of-plane magnetic field yields a broadening of the *Q*-pockets and a reduction of local order. Therefore, the entropy of the potential spin configurations is increased along the high symmetry axes in an applied magnetic field. The origin of this effect is most likely due to magnetostriction, as was shown in ref. ^49^. Hence, to induce dynamics and subsequent aging, we perturbed the self-induced spin glass state by cycling an applied magnetic field. Specifically, we imaged a snapshot of the real-space magnetization in the absence of an applied magnetic field at *T* = 1.3 K (Fig. 3a), cycled the magnetic field to a given value, and reimaged the same area once the field was removed (Fig. 3b), freezing the dynamics back out. This allows us to image snapshots of **M**(**r**, *t*) at subsequent instances (see “Methods” for more details).

### Aging and dynamic heterogeneity after magnetic field cycling

In order to claim that subsequent images are related to aging, it needs to be shown that consecutive snapshots of **M**(**r**, *t*) depend on the previous state. Namely, consecutive snapshots need to contain information about the evolution in phase space, i.e., there is a finite overlap between snapshots. In this way, the evolution depends partially on the history of the sample and can be related to aging. To fully determine phase space, it would be necessary to identify both the magnetization and its dynamics for each spin site in the statistical ensemble at all moments in time. This is not experimentally feasible: consecutive snapshots are taken with many hours lapsing. Furthermore, there are still no experimental methods available that are capable of imaging the magnetization with atomic-scale resolution as well as the magnetization dynamics with femtosecond resolution, especially at the necessary temperatures^50^.

To demonstrate that **M**(**r**, *t*) contains phase space information, we experimentally studied three cases as shown in Figs. 3 and S3: (i) imaging of the same area at *T* = 1.3 K with no applied perturbation (Fig. 3d, e), (ii) imaging of the same spatial region after thermal cycling above/below *T*_g_ (Fig. 3g, h) and (iii) imaging after magnetic field cycles (as shown in Fig. 3a, b). In the case of (i), we observed nearly identical consecutive snapshots. Based on a random walk argument (see ref. ^48^), the return probability for the ensemble is negligible in the case that we consider single spin flips for all sites. Hence, it is statistically improbable that the individual sites or a set of coupled sites evolved through phase space and recovered the same local order over the whole area. Accordingly, we can argue that these regions are frozen out and that we gain full information about the history of the sample and its phase space (Fig. 3f). In contrast, for case (ii), we see insignificant overlap between images when we imaged a given area, thermally cycled it above the *T*_g_, and reimaged the same area. In this case, we lose all information about the history between snapshots, as illustrated in Fig. 3i. For case (iii), when we apply a magnetic field cycle using amplitudes *B* < 3 T, we observe that a significant part of the measured area remains unchanged (see Fig. 3a, b). This indicates that there is partial information about the history of the sample, as indicated in the phase space picture in Fig. 3c. This assumes that sufficiently large regions are highly improbable to fluctuate and return to the same configuration, i.e., they most probably remain frozen through the perturbation. Therefore, the various snapshots of **M**(**r,** *t*) can be related to the aging dynamics of the self-induced spin glass, as we maintain partial phase space overlap, i.e., there are correlations between snapshots.

**Fig. 4:**
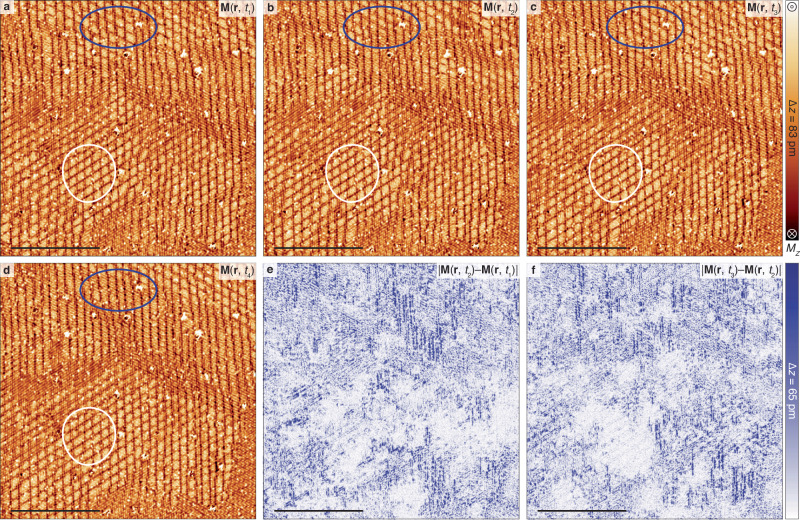
Snapshots of **M**(**r**, *t*) and dynamic heterogeneity. **a** Magnetization image **M**(**r**, *t*_1_) measured at *T* = 1.3 K and *B* = 0 T, where the dynamics are frozen. **b**–**d** In order to observe DH, dynamics are induced through magnetic field cycles from *B* = 0 T to *B* = 2 T (*B* = 4 T for (**d**)), and back. By repeated field cycling and subsequent imaging, snapshots of the dynamics are obtained: **M**(**r**, *t*_i_) after the *i*th field cycle. White (blue) circles mark regions where local magnetic patterns are stable (changing) to illustrate a coexistence of slow and fast dynamics. **e**, **f** Subtracted magnetization snapshots |**M**(**r**, *t*_*i *+ 1_) − **M**(**r**, *t*_*i*_)| suggest spatial regions that change (blue) while others remain the same (white), but also show the complexity of the multi-*Q* dynamics (image parameters: *I*_t_ = 200 pA; scale bar **M**(**r**): 50 nm, scale bar **S**(**Q**): 3 nm^−1^, color scale for **S**(**Q**)-image is a linear gradient, scale bar subtraction images: 50 nm; defect density: 0.0045 ML).

In the subsequent discussion, we studied snapshots of **M**(**r**, *t*) after cycling the magnetic field, such that there was nonzero overlap between subsequent images. In this way, the magnetic field cycling mimics annealing the self-induced spin glass state, and we can relate the snapshots to aging. In Fig. 4a–d, we illustrate four magnetization images of an identical area of 144 × 144 nm^2^ after subsequent perturbations with magnetic field cycles to *B* = 2–4 T. We focus our analysis on images taken after the first field cycle to ensure that **S**(**Q**, *t*) is nearly time-independent (see Supplementary Section S4). As exemplified by the white/blue circles, there are spatial regions in which the local order is frozen/changes, respectively. This coexistence of both similar and dissimilar regions indicated the presence of heterogeneous dynamics, namely slow (no change) and fast (change) dynamics linked to particular areas in real space. To corroborate this, we subtracted subsequent magnetization images to illustrate the local changes between consecutive time steps: **M**(**r**, *t*_2_) − **M**(**r**, *t*_1_) (Fig. 4e) and M(**r**, *t*_3_) − **M**(**r**, *t*_2_) (Fig. 4f). There are two distinct types of behavior in the subtracted images: (a) regions where the intensity is nearly zero. This indicated that these areas exhibited nearly no change, i.e., slow dynamics, between the snapshots. (b) Regions where the intensity is far from zero. These regions changed during subsequent images, indicating the presence of faster dynamics in comparison to the slow areas. This behavior persisted for continued cycling (Supplementary Fig. S5). We did not observe the growth of a persistent pattern, as would be expected for the growth of a favorable domain. We also did not find significant differences when lowering the magnetic field sweep rate by an order of magnitude. While such a subtraction analysis suggests the presence of DH and indicates that the changes are not random, it does not quantitatively distinguish how local areas change in subsequent images.

### Quantifying DH using a wavelet analysis

**Fig. 5:**
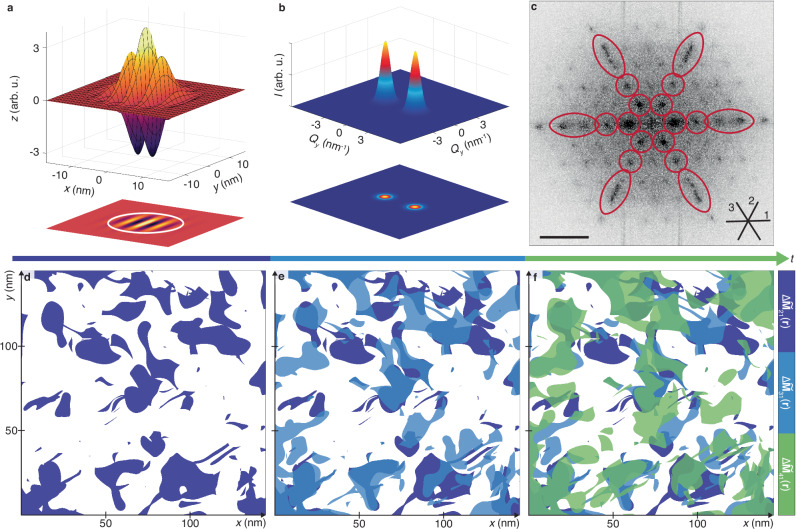
Wavelet quantification of dynamic heterogeneity. A typical Morlet wavelet in **a** real space and **b** Fourier (*Q*) space, illustrating its finite extent in both. **c** The different wavelets used for the subsequent analysis to sample each *Q*-pocket individually (for each of the three *Q*-pockets and for each of the three high symmetry directions labeled 1, 2, and 3). Contours in red contain 90% of the wavelet intensity in *Q*-space and are overlaid over **S**(**Q,** *t*_1_). **d**–**f** Sequence of wavelet filtered images $\Delta \widetilde{{{\bf{M}}}}$_*ij*_(**r**) comparing $\widetilde{{{\bf{M}}}}$(**r,** *t*_*i*_) to the reference $\widetilde{{{\bf{M}}}}$(**r,** *t*_*j*_): **d** $\Delta \widetilde{{{\bf{M}}}}$_21_(**r**) shows a coexistence of slow (white) and fast (blue) dynamics. **e** $\Delta \widetilde{{{\bf{M}}}}$_31_(**r**) overlayed over $\Delta \widetilde{{{\bf{M}}}}$_21_(**r**) illustrates a spatial correlation between subsequent time steps. **f** Evolution of regions of fast dynamics between three consecutive time steps demonstrates persistence behavior and DH in neodymium.

To better quantify the emergence of local length scales associated with particular dynamics, it is essential to gain information about the various periodicities observed in real space. To do this, we adopted the concept of a wavelet transformation (“Methods”), which provides both real space and reciprocal space information simultaneously with limited resolution, similar to Fourier transformations. A wavelet transform compares the signal of interest with a chosen analyzing wavelet, whose frequency and amplitude modulation can be controlled. We implemented a wavelet transform based on a Morlet wavelet. As displayed in Fig. 5a, a Morlet wavelet is an anisotropic waveform of finite size in both real and Fourier space (Fig. 5b). Wavelet convolution is similar to Fast Fourier transform and its inverse (iFFT). However, methods using filtered FFTs and extracting iFFTs can traditionally suffer from artifacts due to how the FFT is filtered, e.g., truncating sine waves. In contrast, the finite size of a wavelet is advantageous in order to correlate real and reciprocal space information, as it allows to quantify the presence of particular periodicities directly in real space. Moreover, a wavelet approach can be tailored to a specific signal of interest, as the wavelet’s shape, angle and scale can be adjusted. This makes it suitable to study multi-*Q* states with a finite distribution in Fourier space, such as the *Q*-pockets observed for Nd(0001), as a subset of *Q*-vectors can be sampled and correlated with its presence in local regions in real space. While the position of the wavelet in *Q*-space determines which periodicities can be sampled, its real-space extent defines its resolution, i.e., the uncertainty on any detected boundary (see Supplementary Section S6). Based on this method, we sampled each of the three *Q*-pockets in **S**(**Q**, *t*) individually with a specific wavelet (Fig. 5c). We then computed its convolution with **M**(**r**, *t*), which we refer to as the wavelet image$\,\widetilde{{{\bf{M}}}}$(**r**, *t*), in order to delineate, where *Q*-pocket periodicities are present in real space (detailed in Supplementary Section S7). We calibrated the wavelet-based method on the long-range ordered state (ref. ^47^) observed at *T* > *T*_g_ = 8.1 K (Supplementary Sections S8 and S9).

The difference ${\Delta \widetilde{{{\bf{M}}}}}_{{ij}}$(**r**) $\propto \widetilde{{{\bf{M}}}}$(**r**, *t*_*i*_) -$\widetilde{{{\bf{M}}}}$(**r**, *t*_*j*_) between subsequent wavelet images $\widetilde{{{\bf{M}}}}$(**r**, *t*_*i*_) compared to the reference image $\widetilde{{{\bf{M}}}}$(**r**, *t*_*j*_), obtained from the different snapshots of Fig. 4, is shown in Fig. 5d–f. For each image, we sampled the individual *Q*-pockets illustrated in Fig. 5c and considered the uncertainty related to the finite wavelet size (see Supplementary Section S10). ${\Delta \widetilde{{{\bf{M}}}}}_{{ij}}\left({{\bf{r}}}\right)$ quantitatively shows the evolution and distribution of fast dynamics in real space and therefore enables the tracking of specific real-space regions and correlating its subsequent dynamics. In Fig. 5d, the blue colored regions illustrate the presence of specific regions in real space that have changed, thus demonstrating a coexistence of slow and fast areas. Tracking these regions after the next field cycle (Fig. 5e) reveals that there is again a coexistence of different time scales, but also that the evolution is not completely randomized in real space. Compiling multiple sequences together (Fig. 5f) illustrates that fast dynamics persistently emerged in similar spatial locations, while at the same time, there were also stable regions without changes (slow dynamics). This demonstrates that the dynamical evolution of **M**(**r**, *t*) is spatially correlated, which holds true also after further cycling (Supplementary Section S11). We found that the percentage of regions characterized by fast dynamics compared to the total measured area increases over time, starting off at 29 ± 2% for $\Delta \widetilde{{{\bf{M}}}}$_21_(**r**), rising to 32 ± 2% for $\Delta \widetilde{{{\bf{M}}}}$_31_(**r**), and 39 ± 2% for $\Delta \widetilde{{{\bf{M}}}}$_41_(**r**) (see Supplementary Fig. S12a). For further field cycling, the percentage of fast regions reaches close to 50%, as can be seen in Supplementary Fig. S12b.

### Effect of defect distribution on DH

In order to claim the observation of DH, it is crucial to disentangle and identify the role of local defects on the dynamical evolution of the magnetization. The most direct result of the presence of defects is defect pinning, which can potentially influence and ultimately quench the dynamics of particular sites locally. To study the role of defects as potential pinning centers, we performed different experiments: (i) we quantified the observation of DH for different samples with varying defect densities in the range of 0.0039 ML to 0.0074 ML, where 1 ML corresponds to the atomic surface density of Nd(0001); (ii) we quantified the spatial correlation between regions that show DH and the location of various defects identified in images, (iii) we studied an identical area and quantified DH between thermal cycles, i.e., randomizing the self-induced spin glass state, to see if the spatial regions observed reoccur in the same location (i.e., defect pinning).

**Fig. 6:**
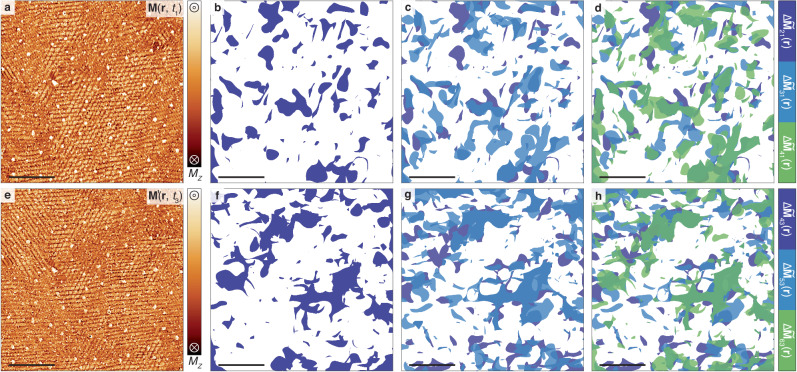
Spatial distribution of fast regions for different thermal cycles. DH for the same area for two different thermal cycles: **a** magnetization image **M**(**r**, *t*_1_) of a sample with defect density of 0.0068 ML after thermal cycling above/below *T*_g_ and the first magnetic field cycle to *B* = 2 T. **b**–**d** Sequence of wavelet filtered images $\Delta \widetilde{{{\bf{M}}}}$_*ij*_(**r**) comparing $\widetilde{{{\bf{M}}}}$(**r,** *t*_*i*_) to the refe**r**ence $\widetilde{{{\bf{M}}}}$(**r,** *t*_*j*_): **b** $\Delta \widetilde{{{\bf{M}}}}$_21_(**r**) shows a coexistence of slow (white) and fast (blue) dynamics. **c** $\Delta \widetilde{{{\bf{M}}}}$_31_(**r**) overlayed over $\Delta \widetilde{{{\bf{M}}}}$_21_(**r**) illustrates a spatial correlation between subsequent time steps. **d** Evolution of $\Delta \widetilde{{{\bf{M}}}}$_*ij*_(**r**) over three consecutive time steps demonstrates the persistence of fast dynamics in specific spatial regions. **e** Magnetization image ${{\bf{M}}}^{\prime}$(**r**, *t*_3_) on the same area after thermal cycling above/below *T*_g_ and the third magnetic field cycle to *B* = 7 T. **f**–**h** Sequence of wavelet filtered images $\Delta \widetilde{{{\bf{M}}}}$_*ij*_(**r**): **f** $\Delta \widetilde{{{\bf{M}}}}$_43_(**r**) shows the appearance of fast dynamics in different regions in space compared to (**b**). **g** $\Delta \widetilde{{{\bf{M}}}}$_53_(**r**) overlayed over $\Delta \widetilde{{{\bf{M}}}}$_43_(**r**) illustrates a spatial correlation between subsequent time steps. **h** Evolution of regions of fast dynamics between three consecutive time steps demonstrates that DH is not driven by defect pinning (image parameters: *I*_t_ = 200 pA; scale bar: 50 nm).

Related to (i), we found similar behavior on four different samples with different defect densities (Supplementary Section S13), where slightly different **S**(**Q**, *t*) were favored, illustrating that DH is prominent regardless of the variations from sample to sample. In all the cases we studied, we saw similar behavior of fast regions appearing randomly in space and persisting in similar spatial regions for subsequent field cycles, irrespective of the exact defect density within the above-mentioned range. Next (ii), we checked if the emergence of regions characterized by different dynamics was correlated with the position of defects or clusters of defects. In particular, we identified the location of defects in the magnetization images and compared their position with the presence of slow and fast regions as determined by the wavelet analysis. We did not see a clear real-space correlation between the occurrence of slow/fast dynamics and the location of any given distribution of defects (Supplementary Section S14). Lastly (iii), we investigated DH in the same area for two different instances, where we thermally cycled the sample by heating it above the glass transition temperature *T*_g_ and cooling it back into the self-induced spin glass phase to *T* = 1.3 K. Figure 6a shows a magnetization image **M**(**r**, *t*_1_) of a sample with a defect density of 0.0068 ML after the first thermal cycle and the initial magnetic field cycle to *B* = 2 T. We obtained a sequence of three wavelet-filtered images $\Delta \widetilde{{{\bf{M}}}}$_*i*1_(**r**) with respect to the reference image $\widetilde{{{\bf{M}}}}$(**r**, *t*_1_). Figure 6b–d shows that fast dynamics appear in particular regions in real space and persist for continued cycling, reminiscent of DH. We then thermally cycled the sample again. This randomized the spatial locations of the different magnetic patterns in the area (see e.g. Fig. 3g, h). We then repeated the magnetic field cycling procedure to *B* = 7 T and performed the wavelet analysis using ${{\bf{M}}}^{\prime}$(**r**, *t*_3_) (Fig. 6e) as a reference. From Fig. 6f–h, it can be seen that the spatial distribution of fast dynamics after thermal cycling significantly differs from the one obtained after zero-field cooling (Fig. 6b–d), while the defect distribution remained the same. Hence, the observation of two different distributions of fast dynamics in the identical area demonstrates that DH is not driven by defect pinning and instead is an intrinsic property of elemental neodymium.

### Length scale of DH and multi-*Q* nature of the local patterns

The demonstration of correlations between regions in real space and their dynamical evolution provides evidence for DH in the self-induced spin glass state of neodymium. In order to characterized DH in more detail, we attempted to identify a particular length scale of DH and its time dependence. This remains challenging due to the arbitrary shapes of the regions possessing fast dynamics, along with their broad size distribution. We calculated a so-called gyration radius, i.e., the average distance from a point in a specific connected cluster of fast dynamics in the $\Delta \widetilde{{{\bf{M}}}}$_*ij*_(**r**)-images to its center of mass, as well as a type of correlation function denoting the average distance between two points within a connected region of fast dynamics in $\Delta \widetilde{{{\bf{M}}}}$_*ij*_(**r**) (see Supplementary Section S15 for details). We found that the correlation function increases over time from around ~16 to ~25 nm (see Fig. S15e). However, DH in neodymium is not fully described by such a single length scale, as can be seen by the broad distributions of gyration radii of the various clusters of fast dynamics present in a given area (Fig. S15a–d). This complexity is likely related to the multi-*Q* nature of the spatially varying magnetic patterns present in the self-induced spin glass state of elemental neodymium. DH has been extensively studied in amorphous materials using other analysis techniques, for example, a real-space four-point correlation function^36,37^. This function, together with its associated generalized susceptibility of the ensemble, is used to quantify the typical size of dynamically correlated regions^38–40^. However, it is challenging to apply this concept directly to the magnetic patterns in neodymium, as it is unclear how to link the multi-*Q* nature of the local spin-spin correlation to the conventional definition of a four-point correlation function.

**Fig. 7:**
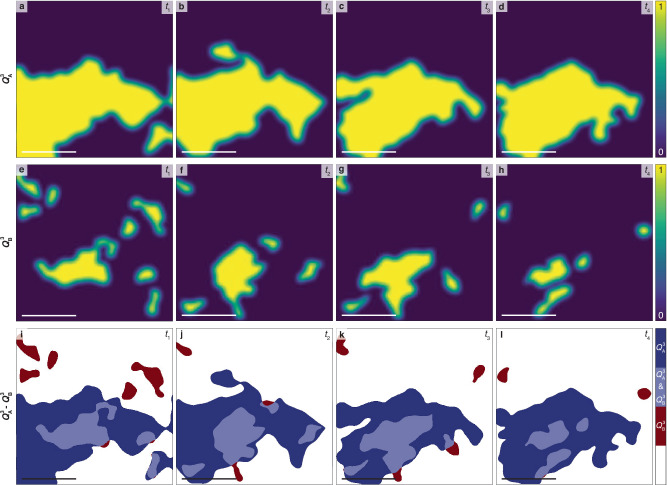
*Q*-resolved wavelet analysis of aging dynamics. Wavelet filtered images $\widetilde{{{\bf{M}}}}$(**r,** *t*_*i*_) for four consecutive time steps *t*_*i*_ corresponding to the images in Fig. 4a–d for two different *Q*-pockets: **a**–**d** ${Q}_{{{\rm{A}}}}^{3}$ and **e**–**h** ${Q}_{{{\rm{B}}}}^{3}$ along the high symmetry direction 3 (see inset Fig. 5c). The real space areas associated with the different *Q*-pockets change over time. **i**–**l** Overlaying $\widetilde{{{\bf{M}}}}$(**r,** *t*_*i*_) for the different *Q*-pockets and time steps (neglecting the uncertainty on the boundary) illustrates that either one (dark blue/red), none (white) or both (light blue) pockets are present in different spatial locations, which likely affects the dynamics (scale bar: 50 nm).

In our analysis, we focused on quantifying changes in periodicities of the three different *Q*-pockets separately. The use of a wavelet analysis allows a deeper investigation into potentially correlating the evolution of various regions with particular *Q*-values (see Fig. S16). Such an analysis may yield further information about the *Q*-dependent favorability of certain *Q*-states, or other thermodynamic processes. We found that a *Q*-resolved analysis is not necessarily straightforward to interpret the physics of the glassy states, due to its multi-*Q* nature. To illustrate this, we performed a qualitative study: we decomposed two different *Q*-pockets using the wavelet analysis for different snapshots, as shown in Fig. 7a–h. The changes in the total area of each *Q*-pocket may be related to how its favorability changes in response to magnetic field cycling. However, this statement may only be made if the dynamics of a single *Q* would be independent of the other possible *Q*-values, i.e., when we have single-*Q* patterns. However, this is not the case for neodymium, as multiple *Q*-pockets are often present in the same location, as can be seen in the example in Fig. 7i–l, where the spatial overlap is moreover time-dependent. This indicates that the dynamics of the different *Q* possibilities are coupled, i.e., that the local order is multi-*Q*-like with multiple possible combinations of *Q*-states. We identified >30 patterns in the films studied. Therefore, the proper treatment of understanding the spatiotemporal evolution with a wavelet analysis of the self-induced spin glass state would require the identification of all the various patterns.

### Atomistic spin dynamics simulations of DH

**Fig. 8:**
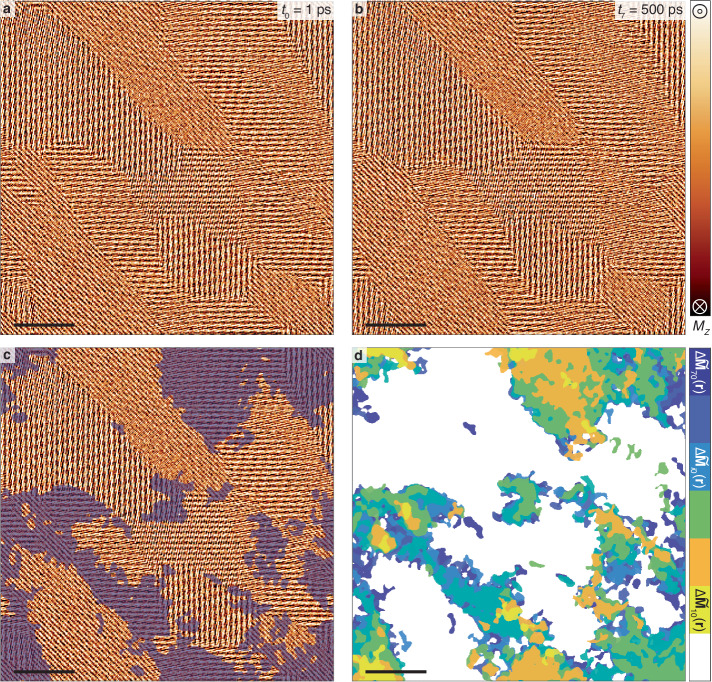
Dynamic heterogeneity and persistence behavior in atomistic spin dynamics simulations. **a** Simulated magnetization image at *t*_0_ = 1 ps showing local magnetic patterns of multi-*Q* character. **b** Magnetization image at a later time *t*_7_ = 500 ps. **c** Map of regions that have evolved over the simulated time (blue) overlayed over the image at *t*_0_ as a reference, revealing spatially heterogeneous dynamics. **d** Regions showing fast dynamics $\Delta \widetilde{{{\bf{M}}}}$_*i*0_(**r**) as a function of time (image at time *t*_i_ compared to initial state at *t*_0_, Δ*t* = 70 ps) indicate a coexistence of slow (white) and fast (colored) regions that nucleate randomly and then grow over time (scalebar: 20 nm).

In order to further substantiate the experimental observation of DH, we performed atomistic spin dynamics (ASD) simulations in which we compared subsequent snapshots of **M**(**r**, *t*). For illustrative purposes to study larger lateral regions, we have used a different simulation cell for the ASD simulations in this work, namely 300 × 300 × 4 unit cells, compared to 32 × 32 × 32 unit cells used in ref. ^46^. We subsequently studied the real-space magnetization as a function of time. Figure 8a shows such a simulated real-space magnetization image at an initial time *t*_0_ = 1 ps (see Supplementary Fig. S17 for a decomposition into the two magnetic sublattices and Supplementary Section S18 for more details). The magnetization revealed multiple types of local magnetic order of multi-*Q* character, without any favorable long-range multi-*Q* state. We then tracked the evolution of **M**(**r**, *t*) over time and show in Fig. 8b one snapshot during the evolution after *t* = 500 ps. Clearly, particular regions in the image had different patterns compared to Fig. 8a. Simultaneously, there were areas in the image that had not changed. To illustrate these changes, we used a subtraction method between the two images (Supplementary Section S19), to highlight the regions that had changed compared to those that had not (Fig. 8c). This demonstrated again the presence of a spatial distribution of slow and fast dynamics. However, based on these two images alone, it cannot be substantiated if these fluctuations were due to random switching or if there was a systematic dynamical evolution of **M**(**r**, *t*) linked to specific regions in real space.

We subsequently illustrated the evolution of the magnetization at various time steps (Δ*t* = 70 ps) to show persistence behavior associated with DH. In Fig. 8d, we identified and color-coded the regions of change ∆**M**_*i*0_(**r**) (subtraction method in Supplementary Section S19), comparing each consecutive time step *i* with the initial state **M**(**r**, *t*_0_). The image showed that the evolution of fast dynamics in space and time was neither consistent with stochastic behavior (i.e., random switching) nor with the clear movement of a favorable domain. It rather revealed the emergence of regions with particular dynamical behavior. Specifically, fast dynamics nucleated in different spatial locations and subsequently grew outwards over time. Notably, this nucleation is not driven by defect pinning, as defects were not considered in the simulation. The observed persistence behavior clearly demonstrated DH in neodymium in accordance with the definition of Fig. 1a, b and corroborated the experimental findings. In addition, the percentage of regions characterized by fast dynamics was found to increase over time (Fig. S12c), similarly to the experiment. We note that the time scales of the calculation and the experiments are different. However, both show qualitatively the same behavior, namely that there are persistent spatial regions of slow and fast dynamics and thus DH.

We observed analogous behavior for all investigated samples (Supplementary Section S20), indicating that the development of dynamically correlated regions was inherent to elemental neodymium. This is reminiscent of DH seen in structural glasses, where particles with similar mobilities cluster together in space, and the relaxation times among these different regions can vary by orders of magnitude^44,51^. Accordingly, the self-induced spin glass state of neodymium possesses particular length scales inherent to both the real-space structure as well as the dynamics of the magnetization, unlike the expected random uniformity linked to the mean-field description of spin glasses.

We have studied, both experimentally and theoretically, DH in the self-induced spin glass state of Nd(0001). This observation of DH demonstrates that this ubiquitous phenomenon seen in amorphous materials also exists in a spin glass system, necessitating descriptions beyond the mean-field limit. It creates a natural bridge between the two different paradigms. Moreover, it suggests that local regions may age toward equilibrium at different rates, as theoretically explored in studies on nonlocal aging^32,52^, and that local length scales have to be taken into account to construct a mathematically rigorous theory of nonequilibrium spin glass dynamics^53^. It remains to be seen how the spatiotemporal dynamics and the specific length scales introduced in the problem are linked to the favorability of the various periodicities (*Q*-states) in the material. It would also be interesting to compare how these local spatiotemporal dynamics are linked to ensemble measurements, for example, near the phase transition^31^. There remains an open question as to what the physical origin of DH is in Nd(0001). It may result from the underlying exchange interactions, which have a finite extent, yielding a ruggedness to the energy landscape. In addition, spatially resolved measurements of the temperature dependence of DH, as well as the influence of temperature variations, would give valuable insight into how spin glasses are able to show memory effects. This latter point may be useful in schemes related to material-based realizations of the Hopfield network and the Boltzmann machine^48,54,55^.

## Methods

### Experimental methods

Nd(0001) islands were epitaxially grown on a cleaned W(110) substrate using the Stranski–Krastanov mode (see ref. ^46^ for details on sample growth). The thicknesses of the four studied islands were between 70 and 180 ML and had a lateral size of at least 300 nm, making these islands bulk-like^46^. The experimental studies were performed in a home-built, ultra-high vacuum system, which operates at a base temperature of 1.3 K with magnetic fields up to 9 T perpendicular to the sample. The SP-STM measurements were acquired with an antiferromagnetic Cr tip prepared with out-of-plane spin-polarization. Magnetization images were produced by subtracting the majority (*V*_S_ = −150 mV) and the minority (*V*_S_ = 200 mV) SP-STM images, as shown in Supplementary Fig. S1 (detailed description in ref. ^46^), and corresponding **S**(**Q**)-images were produced by computing the FFT. The image data processing was performed using MATLAB.

We adopted the following procedure to obtain snapshots of the aging dynamics: (I) a given sample was zero-field cooled below its Néel temperature (*T*_N_ = 19.9 K), through its long-range ordered multi-*Q* phase^47^, and subsequently below the glass transition temperature (*T*_g_ ~ 8 K) down to *T* = 1.3 K. At *T* < *T*_g_, long-range magnetic order dissolved into locally ordered magnetic patterns, which exhibited complex aging dynamics^46,47^. These dynamics froze out at low temperatures, and the various frozen patterns could be imaged at *T* = 1.3 K (and *B* = 0 T). At this temperature, no substantial changes in the magnetization over the course of 27 h were observed (see Supplementary Fig. S2). (II) We applied magnetic field cycles to *B* = 2–4 T to induce magnetization dynamics during the *B*-field sweep^46^. The dynamics were frozen again when the applied field was removed, and the resulting evolved state could be re-imaged. Hence, by repeating step (II) we obtained a series of snapshots of the aging dynamics as displayed for example in Fig. 4a–d. The sweep rates we used were 3.7 mT/s from 0 to 3.7 T, 3 mT/s from 3.7 to 5.6 T and 1.85 mT/s from 5.6 to 9 T. We typically applied the maximum field during a field cycle for 10 min.

### Wavelet analysis

The wavelet method used for the analysis of Fig. 5 was based on a continuous wavelet transformation (CWT) using the cwtft2 function in MATLAB. The cwtft2 function computes the 2-D Fourier transforms of the input image and analyzing wavelet, which are then multiplied together and inverted. We used the predefined Morlet wavelet in MATLAB. Generally, a CWT will be a function of both position and scale of the wavelet (compressed or stretched versions of the wavelet), but we fixed the scale of the wavelet to independently probe the *Q*-pockets. A general introduction on wavelet transformations can be found in refs. ^56,57^.

### Theory

All simulations were performed using the ASD software UppASD^58^, with full algorithmic details provided in ref. ^59^. The dhcp Nd system was modeled using the same spin Hamiltonian as described in ref. ^46^, specifically a Heisenberg Hamiltonian parameterized with scalar Heisenberg exchange interactions derived from density functional theory (DFT). No anisotropic contributions, such as on-site magnetocrystalline anisotropy or anisotropic exchange interactions, were included in the Hamiltonian. The magnetization snapshots presented in Fig. 8 were obtained by simulating a slab containing 300 × 300 × 4 dhcp unit cells, and periodic boundary conditions were assumed in the *x* and *y* directions. To ensure that the flat geometry considered in this work did not alter the dynamics earlier observed for thicker samples^46^, we confirmed by studies of the two-time autocorrelation function (see Fig. 1e and Supplementary Section S21 for results and details) that these systems reproduce the glassy behavior as reported in ref. ^46^.

## Supplementary information

Supplementary Information

Transparent Peer Review File

## Data Availability

The data generated in this study have been deposited in an open-access database under accession code 10.34973/hs52-vj57.

## Code availability

The codes that support the findings of this study are available from the corresponding author upon reasonable request.
