# Supplementary material for: Dynamic heterogeneity in the self-induced spin glass state of elemental neodymium
Source: Nat Commun. 2026 Jun 20;17:7787. doi: 10.1038/s41467-026-74637-1 (PMC13439053; doi:10.1038/s41467-026-74637-1)
Supplement: Supplementary file 1 [file 41467_2026_74637_MOESM1_ESM.pdf]

Supplementary Materials for

Dynamic heterogeneity in the self-induced spin glass state of elemental neodymium

Authors: Lorena Niggli[1], Julian H. Strik[1], Karoline Oetker[1], Zhengyuan Liu[1], Anders Bergman[2,3], Nadine Hauptmann[1], Mikhail I. Katsnelson[1], Daniel Wegner[1], Alexander A. Khajetoorians[1]*

*Corresponding author: a.khajetoorians@science.ru.nl

The PDF file includes:

Supplementary Text
References
Figs. S1 to S21

Supplementary Text

S4. Initial evolution of S(Q, *t*) during magnetic field cycling and its saturation
After the initial cool down, a magnetic field cycle to B = 2 T induced substantial changes to the corresponding magnetic structure factor **S**(**Q**, *t*). Figs. S4a-b illustrate a change in the positions of the *Q*-pockets, when comparing **S**(**Q**, *t*) before and after the first application of a magnetic field. This is reminiscent of rejuvenation effects induced by dc magnetic fields in spin glasses (*1*). However, **S**(**Q**, *t*) stabilized to a final distribution after the initial field cycle, as can be seen in Figs. S4b-d. The observations of DH using magnetic field cycles were studied in the limit after **S**(**Q**, *t*) stabilized to its final state. We note that we did not reach the ferromagnetic limit neither (a) by imaging in magnetic fields up to 9 T, nor (b) after continued field cycles (e.g. due to the potential favorability of ferromagnetic domains).

S5. Coexistence of slow and fast dynamics during continued cycling
In Fig. 4, we observed a coexistence of regions exhibiting slow and fast dynamics for the first three field cycles. To demonstrate the presence of heterogeneous dynamics for subsequent cycles, we measured a series of 12 magnetization images corresponding to 11 field cycles to B = 2 T on a different sample with a defect density of 0.0074 ML. Fig. S5 shows **M**(**r**, *t*) from t_4 onwards as well as the corresponding subtracted images |**M**(**r**, t_{i+1}) - **M**(**r**, t_i)|. As can be seen, slow and fast dynamics coexist at all measured times, since each subtraction image showed regions in real space, where the intensity is nearly zero (stable = slow) as well as regions with significant intensity associated with fast dynamics.

S6. Benchmarking and the spatial resolution of the wavelet transformation
In order to benchmark the wavelet approach used in the main paper, we first considered a simulated magnetization image **M**(**r**) (Fig. S6a). We intentionally created two domains described by a single-*Q* state: An inner box exhibited Q_1 = 6 nm^{-1}, whereas the rest of the image exhibited Q_2 = 3 nm^{-1}. These two *Q*-states chosen along different directions can also be seen in **S(Q)** in Fig. S6b. We then defined a specific Morlet wavelet addressing Q_1 with a varying real space diameter (FWHM) ranging between ~2.2 - ~40 nm, which corresponds to the *Q*-space contours displayed in Fig. S6c. Examples of the resulting wavelet transformation images **W**(**r**) for two wavelet sizes are shown in Figs. S6d-e. In both images, the inner box region correctly showed a large intensity compared to the vanishing intensity outside in accordance with the simulated **M**(**r**). However, the identified boundary between the Q_1- and Q_2- regions smeared out as the real-space size of the wavelet increased. This can be seen in Fig. S6f, where line cuts along the white lines indicated in Figs. S6d-e are shown. In the simulation, the size of the inner box could be retrieved from the line profiles by using the half maximum in intensity. Applying this to the spin glass case is more complex: **M**(**r**, *t*) exhibits a broader distribution of different *Q*-areas, with comparatively smaller regions defined by a multi-*Q* character. There are also noise considerations. In order to define a systematic approach to wavelet filtering in this case, we take the finite wavelet size into account using an uncertainty on any detected boundary, which we defined to amount to the FWHM of the real-space wavelet.

S7. Wavelet transformation: calibration using the long-range ordered state and systematic definition of noise threshold
We first applied the wavelet transform to the long-range ordered state observed above the glass transition temperature T_g ~ 8 K, as previously reported in ref. (*2*), in order to characterize the error in the method for the analysis in the spin glass state. Fig. S7a shows a magnetization image measured at T = 11 K and B = 0 T, which is characterized by two

dominant domains of different multi-Q type that are separated by domain walls (indicated by drawn white dashed lines). The corresponding **S**(**Q**) exhibited sharp Q-spots indicative of long-range order, as displayed in Fig. S7b. We defined one Morlet wavelet to address a specific Q-spot (blue circles, containing 90% of the wavelet intensity in Q-space). We then calculated the wavelet transformation **W**(**r**) of this wavelet with the real-space magnetization (see Methods) and show the result in Fig. S7c. As can be seen by the regions of significant intensity (green and yellow) of **W**(**r**), the Q-spot we probed is present in one of the domains, which we confirmed by regular Fourier transformation on a smaller area (Fig. S7d), but not in the other. In order to determine the area, where this specific Q is present in real space, we have to define an appropriate threshold for **W**(**r**). To this end, we calculated a wavelet transformation, where the same wavelet (blue circles in Fig. S7b) is rotated away from the Q-spot to address solely noise (red circles in Fig. S7e). The resulting **W**(**r**) is shown in Fig. S7f and exhibited a random distribution of intensity (the intensity is approximately 10 times smaller than in Fig. S7c). Using this, we defined a systematic noise threshold, where everything below it is considered noise. In instances where we did not measure the magnetization image for the long-range ordered state, we derived the noise threshold by calculating it from a **S**(**Q**)-image in the self-induced spin glass state and adapted the threshold to optimize the connectedness of the filtered **W**(**r**) (used for the sample of Figs. 4 and 5). Fig. S7g shows the wavelet image $\widetilde{\mathbf{M}}(\mathbf{r})$ after thresholding **W**(**r**), which now quantifies the regions in space (yellow), where the associated Q-state is present. As can be seen in Fig. S7h, where we overlayed this identified Q-area over the initial magnetization image, we find a clear correspondence with the different domains. Lastly, as discussed in Supplementary Section S6, the boundaries of the identified Q-area are only defined within an uncertainty related to the wavelet size. To take this into account, we smeared out the filtered wavelet image of Fig. S7g using the FWHM of the wavelet in real space. In this way, the introduced wavelet method allowed us to quantify the regions in real space inhabited by a specific Q-state (Fig. S7i) to characterized their evolution over time.

S8. Quantifying the multi-Q nature of the long-range ordered state to benchmark the wavelet analysis

We applied the wavelet analysis to the long-range ordered state, at $T = 11$ K (Fig. S8a), to bench mark the method. We used multiple distinct wavelets to address the two Q-states (Q_B and Q_C) separately for each high symmetry axis. Fig. S8b shows the **S**(**Q**)-image and overlaid circles containing 90% of the wavelet intensity in Q-space. The calculated wavelet transforms **W**(**r**) are shown in Figs. S8c, e and the corresponding wavelet images $\widetilde{\mathbf{M}}(\mathbf{r})$ in Figs. S8d, f decomposed into the different Q-states. As can be seen from the wavelet images in Figs. S8d, f the identified spatial distributions of the Q-states qualitatively matched the domains and domain walls identified by eye in Fig. S8a. In addition, when the different $\widetilde{\mathbf{M}}(\mathbf{r})$ were overlayed (Fig. S8g), albeit neglecting the uncertainty on the boundaries, we confirmed that the long-range ordered state at $T = 11$ K exhibited multi-Q order, as observed in Ref. (*2*). We also identified the local multi-Q order by separating the area into a grid of boxes of size 4.7 $\times$ 4.7 nm^2. For each box, we checked which Q-states were fully present and then colored each different Q-combination with a specific color. The resulting spatial map of local multi-Q order is shown in Fig. S5h and illustrates the two main domains (blue and yellow) along with a wall region, where the order varies on the nanometer scale. In addition, there were also variations of the order on smaller length scales within the domains likely related to defects as well as artifacts of the chosen global thresholds.

S9. Dependence of the wavelet method on the real-space wavelet size

We compared the wavelet method for three different wavelet sizes using the long-range ordered state at $T = 11$ K (Fig. S9a). The FWHM of the used real space wavelets were as follows: 7.2-7.6 nm^{-1} for Q_B and 4.6-5.9 nm^{-1} for Q_c (smallest), 9.4-10.1 nm^{-1} for Q_B and 7.1-7.8 nm^{-1} for Q_c (middle) and 14.2-15.1 nm^{-1} for Q_B and 13.3-14.4 nm^{-1} for Q_c (largest). The colored circles of the defined wavelets containing 90% of the wavelet intensity in Q-space are shown in Fig. S9b. The corresponding wavelet transforms $\mathbf{W}(\mathbf{r})$ and images $\widetilde{\mathbf{M}}(\mathbf{r})$ are displayed in Fig. S9c-f. $\mathbf{W}(\mathbf{r})$ for the different wavelet sizes consistently revealed significant intensity in the same regions in space indicating the stability of the wavelet method in the chosen size range. However, the resolution and grains of the identified regions of $\widetilde{\mathbf{M}}(\mathbf{r})$ depended on the specific size of the wavelet. Fig. S9d, f illustrate that the method of thresholding can influence the connectedness of the identified Q-areas. We chose the wavelet sizes to optimize both the spatial resolution and the connectedness of the wavelet images $\widetilde{\mathbf{M}}(\mathbf{r})$.

S10. Method to quantify *Q*-resolved changes in the self-induced spin glass state

We outline the procedure of determining the Q-resolved changes of $\mathbf{M}(\mathbf{r}, t)$ to quantify slow and fast dynamics in Fig. 5. As an example, we focused here on one Q_A-pocket addressed by a specific wavelet (Fig. S10b). The associated wavelet images $\widetilde{\mathbf{M}}(\mathbf{r}, t)$ were calculated according to the wavelet method outlined previously. Figs. S10c-d display $\widetilde{\mathbf{M}}(\mathbf{r}, t)$ before and after one field cycle and showed that part of the identified areas related to the probed Q_A-pocket remained the same between the two time steps, while the presence of Q_A-periodicities either appear or disappeared in other local areas. In order to quantify the areas that have changed, we first subtracted the two images $\widetilde{\mathbf{M}}(\mathbf{r}, t_2)$- $\widetilde{\mathbf{M}}(\mathbf{r}, t_1)$ (Fig. S10e) and then considered only the changed regions outside of the uncertainty of the reference image $\widetilde{\mathbf{M}}(\mathbf{r}, t_1)$. The resulting changed areas $\delta\widetilde{\mathbf{M}}_{21}(\mathbf{r})$ associated with fast dynamics between the two time steps for the probed Q_A-pocket are shown in Fig. S10f. We repeated this procedure for all the Q-pockets to quantify and track the total regions $\Delta\widetilde{\mathbf{M}}_{ij}(\mathbf{r})$ exhibiting fast dynamics, which are shown in Fig. 5.

S11. Persistence of DH for further cycles

We studied the time evolution of $\mathbf{M}(\mathbf{r}, t)$ for further iterations to investigate if DH persisted. We applied the wavelet method to the series of 12 magnetization images $\mathbf{M}(\mathbf{r}, t)$ corresponding to 11 field cycles to $B = 2$ T on one sample with a defect density of 0.0074 ML (see also S5). We analyzed subsequent times, i.e. the difference of consecutive wavelet images with t_3 (Fig. S11a) and t_7 (Fig. S11m) as reference images. As can be seen in the corresponding $\Delta\widetilde{\mathbf{M}}_{ij}(\mathbf{r})$-series, DH was present for both cases.

S13. Statistics of DH and influence of the surface defect density

We studied the effect of the surface impurity density on the emergence of DH by investigating three other samples, in addition to the sample in the main manuscript, characterized by a varying defect density in the range of 0.0039 ML to 0.0074 ML (where 1 ML corresponds to the atomic surface density of Nd(0001)). In Fig. S13, we show a series of four magnetization images $\mathbf{M}(\mathbf{r}, t)$ and a representative static magnetic structure factor $\mathbf{S}(\mathbf{Q}, t)$ for the different samples. We consistently observed that there is a variance in the measured $\mathbf{S}(\mathbf{Q}, t_0)$ for different islands, i.e. the characteristic Q_A, Q_B- and Q_C-pockets were present on all samples, albeit their intensity and extent varied from island to island. However, we see comparable distributions of $\mathbf{S}(\mathbf{Q}, t)$ regardless of how we perturb the same island. Below a surface defect density of ~0.015 ML, the presence of defects or unconsidered interactions may change the order of low energy states (i.e. their hierarchy) but will not destroy the self-

induced spin glass phase (*3*). This can lead to a variance between islands but does not modify the observation of DH, as can be seen in the wavelet images $\Delta\widetilde{\mathbf{M}}_{ij}(\mathbf{r})$ and their compilation for the different samples. A similar analysis as done in the main manuscript revealed the same qualitative behavior of the persistence of fast dynamics in specific regions in space reminiscent of DH for all the studied samples. The corresponding percentages of fast dynamics are displayed in Supplementary Figure S12a.

S14. Influence of defects on emergence of slow and fast dynamics
We checked if the emergence of regions characterized by different dynamics was correlated with the position of defects or clusters of defects. To this end, we identified the defects visible on the surface on two different samples characterized by surface defect densities of 0.0045 ML (Fig. S14a) and 0.0039 ML (Fig. S14e), respectively. We marked the position of the defects by blue dots in Figs. S14b and S14f. We note that the distance between defects is considerably smaller than the rough length scale of the local patterns we observe. Figs. S14c and S14g display the identified defect distributions (now red dots) overlayed over the regions possessing slow and fast dynamics for one time step resulting from the wavelet analysis. We see no clear real-space correlation between the locations of defects and the positions of regions that show slow/fast dynamics. This holds for the whole dynamical evolution (Figs. S14d and S14h) and confirms that the experimental observation of DH is not correlated with one specific set of defects.

S15. Length scale of DH
As can be seen in Fig. 5, the identified regions exhibiting fast dynamics possess arbitrary shapes and show a distribution of sizes. We refer to each connected region in a wavelet image $\Delta\widetilde{\mathbf{M}}_{ij}(\mathbf{r})$ as a cluster. In order to quantify a characteristic length scale of DH in neodymium, we analyzed the size of the clusters of fast dynamics by calculating the so-called gyration radius R_s and a correlation length derived from it (see refs. (*4, 5*)). R_s denotes the average distance from a point $\boldsymbol{r}_n$ in a specific cluster of size *s* to its center of mass $\boldsymbol{r}_{\mathrm{cm}}$ and is defined through (*4*)

$$R_s^2 = \sum_{n=1}^{s} \frac{|\boldsymbol{r}_n - \boldsymbol{r}_{\mathrm{cm}}|^2}{s} \quad \text{with} \quad \boldsymbol{r}_{\mathrm{cm}} = \sum_{n=1}^{s} \frac{\boldsymbol{r}_n}{s}$$

Based on the gyration radii, we also computed a correlation length ξ meaning the average distance between two points within a cluster defined as (*4, 5*)

$$\xi^2 = \frac{\sum_s s^2 n_s R_s^2}{\sum_s s^2 n_s}$$

with n_s= (number of clusters of size *s*)/L^2 the cluster number distribution function and L^2 the total number of points (pixels) in the analyzed image. Fig. S15a-d displays the distributions of gyration radii for three consecutive time steps for all the different measured samples characterized by varying defect distributions (see Figs. 5 and S13). Overall, there exist many clusters of fast dynamics with gyration radii below 10 nm along with a small number of clusters exhibiting radii in the range of ~15-38 nm. These broad distributions of R_s demonstrate the complexity of the heterogeneous dynamics observed in neodymium, likely connected to its multi-*Q* nature. Nonetheless, the calculated correlation length ξ (Fig. S15e) increases from ~15-18 nm for the first time step to ~25-35 nm for the third.

S16. *Q*-resolved wavelet analysis in the self-induced spin glass state

The wavelet analysis gives access to the real-space areas for all the different *Q*-pockets present in the self-induced spin glass state. Such a decomposition is shown in Fig. S16 for the sample displayed in the main text (see Figs. 4 and 5).

S17. Decomposition of simulated real-space magnetization into magnetic sublattices

The double hexagonal closed packed crystal structure of Nd(0001) with an ABACA stacking results in two distinct sublattices, which possess different local symmetries: the A layer is sandwiched between two different layers and hence exhibits local cubic symmetry, while the B and C layers are surrounded by the same layer and thus exhibit locally a hexagonal symmetry. They are accordingly referred to as cubic (cub) and hexagonal (hex) sublattices. The effect of these different environments on the exchange interaction was discussed in Refs. (*2, 3*). While the simulated magnetization images in the main text (Fig. 8) display the combined signal of both sublattices, Fig. S17 shows the decomposition of the magnetization into the hex and cub sublattices for t_0 (a-b) and t_7 (c-d) respectively. Both sublattices show short-range order yet with different overall periodicities. Following the local orders for the hex and cub sublattices between t_0 and t_7 reveals spatially heterogeneous dynamics in the form of stable and changing regions similar to the behavior of the total simulated magnetization images shown in Fig. 8 of the main text.

S18. Representation of simulated magnetization textures

Figure 8 in the main text as well as Figs. S17 and S20 display simulated magnetic configurations for dhcp Nd represented as false-color images. The configurations represented in these figures consist of 300 × 300 × 4 unit cells of Nd, with a hexagonal simulation box. To simplify the analysis, we have cropped the area and plotted the magnetic textures in a square representation. This representation corresponds to a coordinate system expressed in the lattice vectors of the actual system and has the benefit that the magnetic texture can readily be expressed on a uniform grid or as elements in a square matrix. As a result of this transformation, all simulated figures display a skewed realization of the real-space spin texture. This linear transformation of the spin textures does not affect the analysis of local dynamics performed in this work but should be noted in case of comparisons with earlier works on the system as done in refs. (*2, 3*). An example on how the square representation transfers to the un-skewed real-space representation can be seen in Fig. S18.

S19. Subtraction method to extract region of change

We applied the following method to quantify regions in the simulated magnetization images that exhibited a change of the local order between a time *t* and the initial time t_0: Fig. S19a-b displays two example magnetization images (a) initial state at time t_0, (b) evolved state at time t_3) that were to be compared in terms of their spin configurations. First, we subtracted the two images (see Fig. S19c), which revealed regions in space where the magnetic contrast approximately vanished. Next, we filtered out those spatial regions possessing a magnetization value close to zero by choosing a threshold that extracts the top 30 % of the extremal magnetization values. The resulting binary image is shown in Fig. S19d and clearly shows regions of accumulated signal as well as regions possessing a lower density of extremal magnetization values. We applied a gaussian filter and another filtering threshold (τ_c = 0.25) to extract the regions of maximal difference in (d) as can be seen in Fig. S19e. In the last step, we filled holes within connected regions using MATLAB's *imfill* function and also neglected the smallest connected regions. Thereby, we obtained a binary image quantifies the regions of change between two time steps (Fig. S19f). We note that this

simplistic analysis does not give information about the nature of the change, i.e. it does not distinguish between a phase shift or a complete change of the local order.

S20. Statistics of simulated local dynamics

In the main text, the magnetization dynamics from a single ASD simulation are used to demonstrate DH and the spatially resolved dynamics in dhcp Nd. To demonstrate that this one simulation is not a statistical outlier or exception, we performed a series of simulations on dhcp Nd for the same system size (300 × 300 × 4 unit cells), where starting states as well as the sequence of random numbers that populate the heat-bath in our Langevin-based simulations (for more details on stochastic ASD simulations see Ref. (*6*)) were varied. Regardless of initial state or random-number streams, we observed similar local dynamics for all simulated samples, as can be seen in Fig. S20. The corresponding percentages of fast dynamics are displayed in Fig. S12c. Different choices of the Gilbert damping parameter were also considered with the same, positive outcome for all cases (data not shown).

S21. Autocorrelation function

In Ref. (*3*), the simulated two-time autocorrelation function

$$C(t, t_w) = \langle \mathbf{m}_i(t_w)\mathbf{m}_i(t + t_w)\rangle \qquad \text{S1}$$

showed glassy behavior for bulk dhcp Nd (with $\mathbf{m}_i$ being the magnetization of site *i*, *t* the time and t_w the waiting time). For illustrative purposes we have used a different simulation cell for the ASD simulations in this work (here we used 300 × 300 × 4 unit cells compared to 32 × 32 × 32 unit cells used in Ref. (*3*)). To ensure that we capture glassy dynamics for the systems simulated here, we calculated the autocorrelation $C(t, t_w)$. For comparison we also simulated an Edwards-Anderson model (EA) for the same 300 × 300 × 4 geometry. These autocorrelation functions are shown in Figs. 1e and S21 and can be compared to previous results in Ref. (*3*). We can conclude that for the systems sizes considered in this study we obtain a similar, but not identical, relaxation behavior for both the dhcp Nd and the EA model systems.

References

1. E. Vincent, J. P. Bouchaud, J. Hammann, F. Lefloch, Contrasting Effects of Field and Temperature-Variations on Aging in Spin-Glasses. *Philos. Mag. B* **71**, 489-500 (1995).
2. B. Verlhac *et al.*, Thermally induced magnetic order from glassiness in elemental neodymium. *Nat. Phys.* **18**, 905-911 (2022).
3. U. Kamber *et al.*, Self-induced spin glass state in elemental and crystalline neodymium. *Science* **368**, eaay6757 (2020).
4. D. Stauffer, *Introduction to percolation theory*. A. Aharony, Ed., (CRC Press, ed. Rev. 2nd., 1994).
5. C. Frydendahl, Fractal analysis package (https://ch.mathworks.com/matlabcentral/fileexchange/71770-fractal-analysis-package). *MATLAB Central File Exchange*, (2026).
6. O. Eriksson, A. Bergman, L. Bergqvist, J. Hellsvik, *Atomistic Spin Dynamics: Foundations and Applications*. (Oxford University Press, 2017).

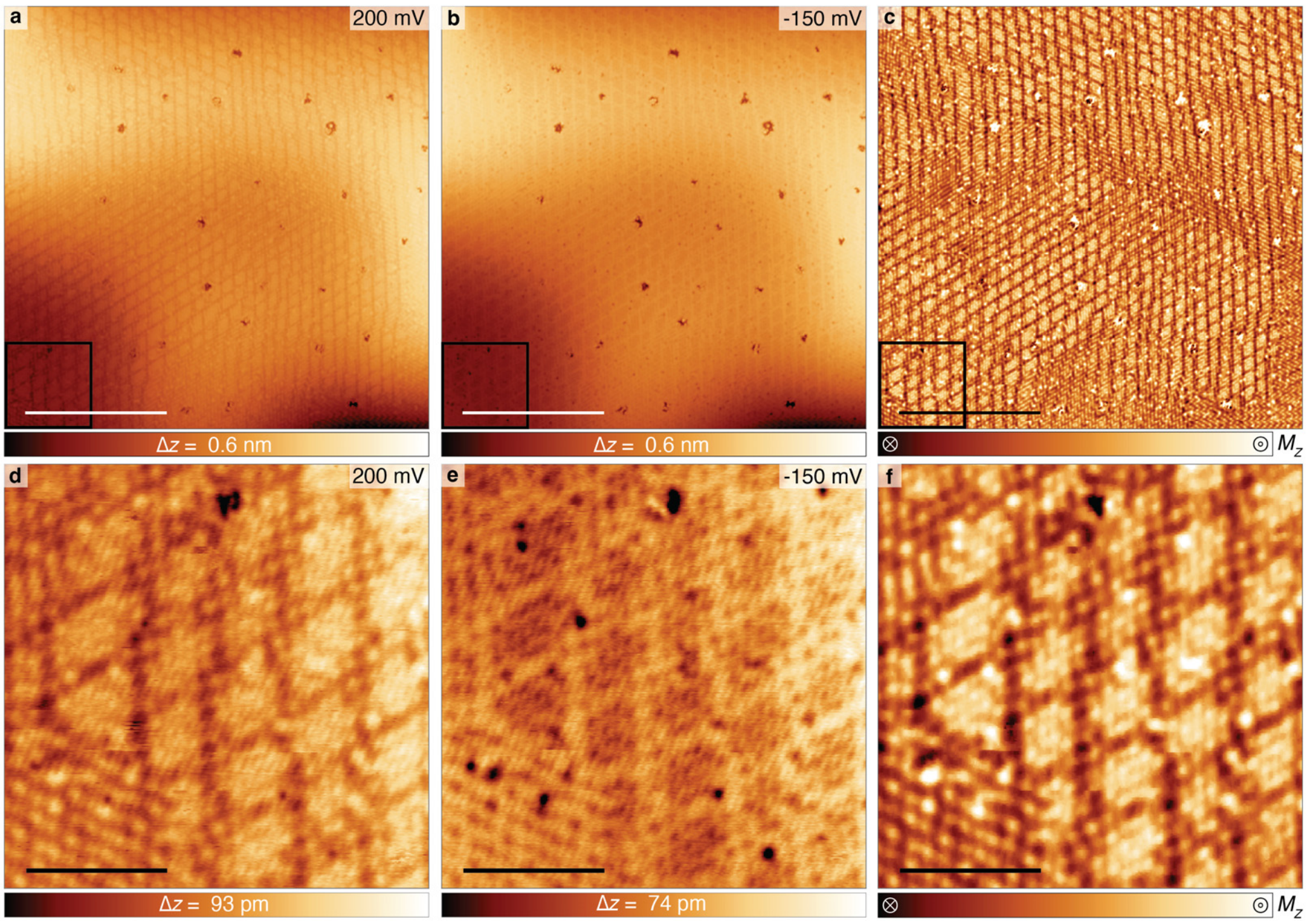

Fig. S1. Magnetization image. (a-b) Large scale topographic images in constant-current mode at two bias voltages, **(a)** at V_S = 200 mV (minority) and **(b)** at V_S = -150 mV (majority), showing both the morphology of the island surface as well as magnetic information (faint stripes). Black outline shows zoom in region for **(d-f)** (I_t = 200 pA, scale bar: 50 nm). **(c)** Magnetization image obtained by subtracting the minority **(a)** from the majority image **(b)**, thereby revealing the magnetic structure of Nd(0001) (scale bar: 50 nm). **(d-e)** Magnetic contrast in the images at V_S = 200 mV **(d)** and V_S = -150 mV **(e)** is reversed. **(f)** Small scale magnetization image obtained from **(d-e)** (scale bar: 10 nm).

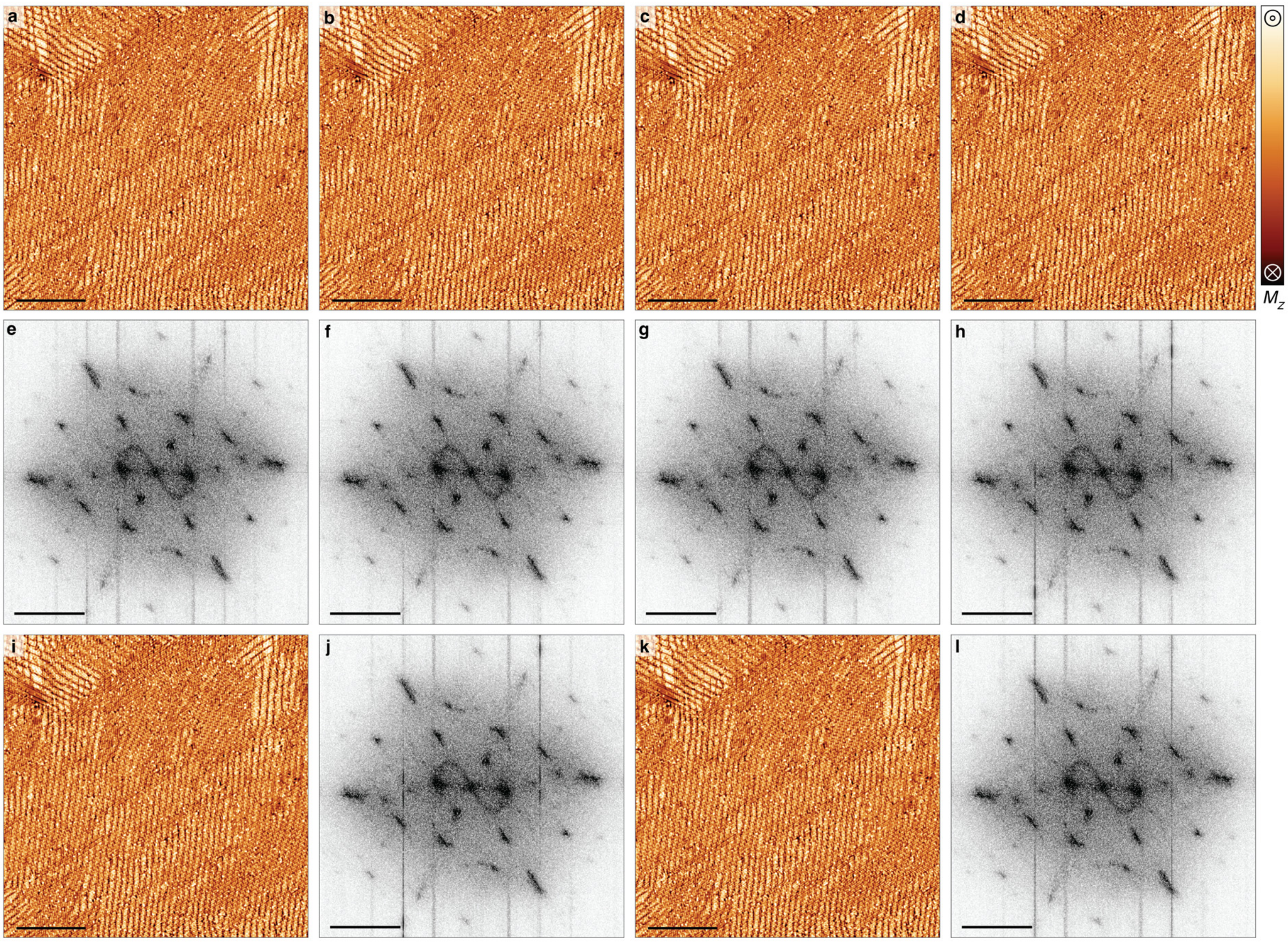

Fig. S2. Frozen real-space magnetization at T = 1.3 K. (a-d, i, k) Large scale magnetization images measured consecutively at T = 1.3 K and B = 0 T (scan direction down, up, down, etc.), where the duration of one image corresponds to ~ 4.5 h, show no change of magnetic patterns over ~ 27 h illustrating frozen out aging dynamics on the experimental time scale. **(e-h, j, l)** **S**(**Q**)-maps corresponding to the images in the row above **(e-h)** or the images to the left **(j, l)** (image parameters: I_t = 200 pA; scale bar for all **M**(**r**) images: 50 nm, colorbar for all **M**(**r**) in the top right, color scale for **S**(**Q**)-images is a linear gradient; defect density of 0.0034 ML).

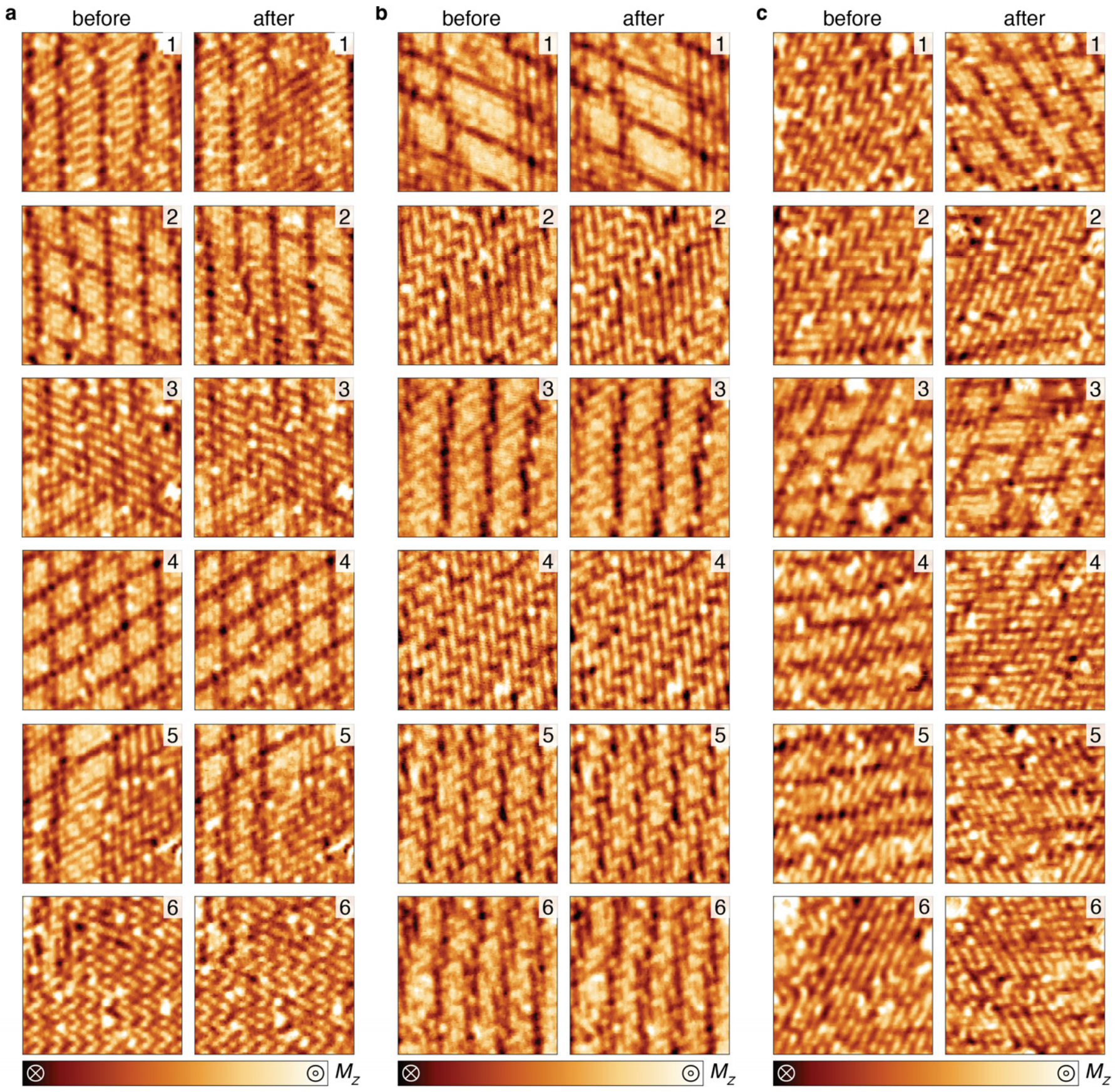

Fig. S3. Influence of perturbation on local multi-*Q* order. Exemplary local areas (corresponding to white boxes outlined and labelled in Fig. 3) for three cases: **(a)** before and after applying a magnetic field cycle to $B = 2$ T, **(b)** before and after consecutively imaging without applying a perturbation and **(c)** before and after heating and cooling across the glass transition temperature ($T_g \sim 8$ K). The local magnetic order **(a)** exhibits both changing and stable patterns, i.e. partial overlap, **(b)** remains frozen and does not change and **(c)** shows next to no correlations before and after re-entering the self-induced spin glass phase (box size: 20×20 nm^2).

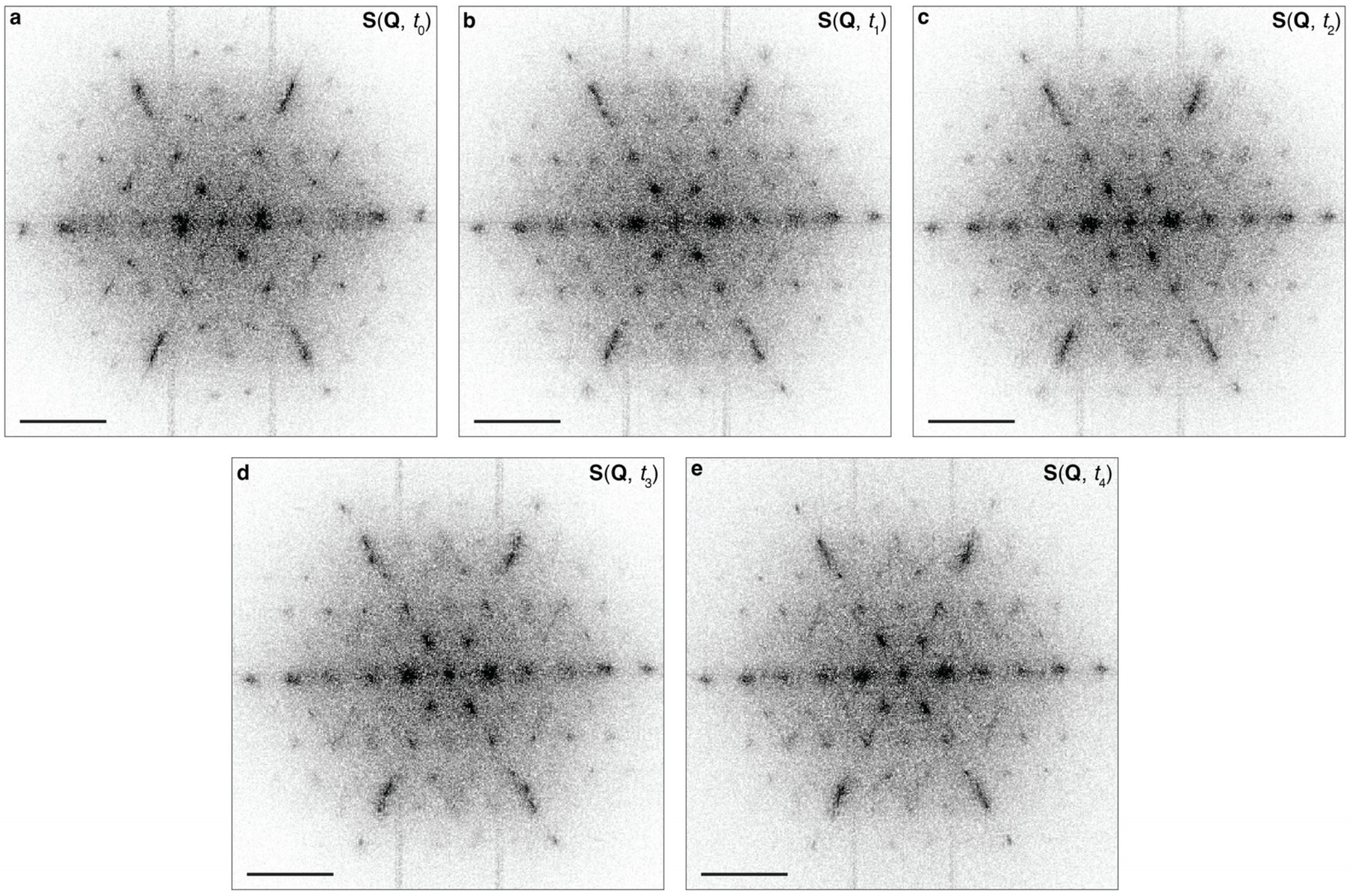

Fig. S4. Time-dependence of S(Q, *t*). S(**Q**, *t*)-images of the same sample as in Fig. 1c, d and Fig. 4: **(a)** S(**Q**, t_0) after zero-field cooling to $T = 1.3$ K characterized by a specific distribution of *Q*-states. **(b)** S(**Q**, t_1) after the first magnetic field cycle to $B = 2$ T reveals that the *Q*-pocket positions changed. **(c)** S(**Q**, t_2) after the second magnetic field cycle to $B = 2$ T exhibits stable *Q*-pocket positions, but with variations of spectral weight within them. **(d)** S(**Q**, t_3) after the third magnetic field cycle to $B = 2$ T is similar to the two preceding ones, i.e. S(**Q**, t_3) ≈ S(**Q**, t_2) ≈ S(**Q**, t_1). **(e)** S(**Q**, t_4) after the fourth magnetic field cycle to $B = 4$ T corroborates the saturation of the *Q*-pocket positions (scale bar: 3 nm^{-1}).

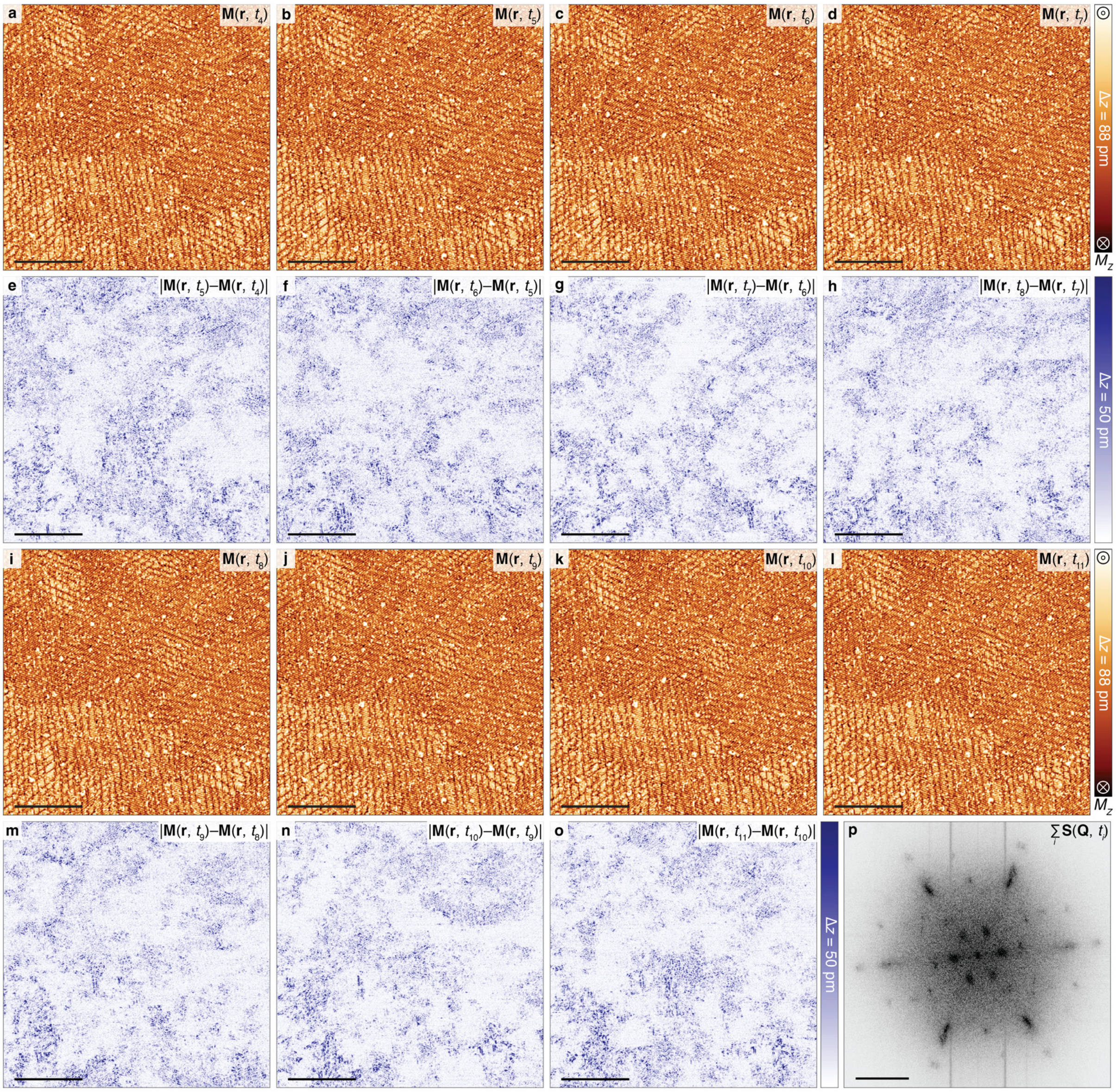

Fig. S5. Slow and fast dynamics in repeated cycles. (a-d, i-l) Magnetization images **M**(**r**, t_i) after the ith field cycle to B = 2T measured at T = 1.3 K and B = 0 T. **(e-h, m-o)** Subtracted magnetization images |**M**(**r**, t_{i+1}) - **M**(**r**, t_i)| reveal spatial regions that changed (blue, fast dynamics) while others remained the same (white, slow dynamics) for all times. **(p)** Individual static magnetic structure factors added together $\sum_i \mathbf{S}(\mathbf{Q}, t_i)$ to illustrate its time-independence (image parameters: I_t = 200 pA; scale bar **M**(**r**) and subtraction images: 50 nm, scale bar **S**(**Q**, t): 3 nm^{-1}; color scale for **(p)** is a linear gradient; defect density of 0.0074 ML).

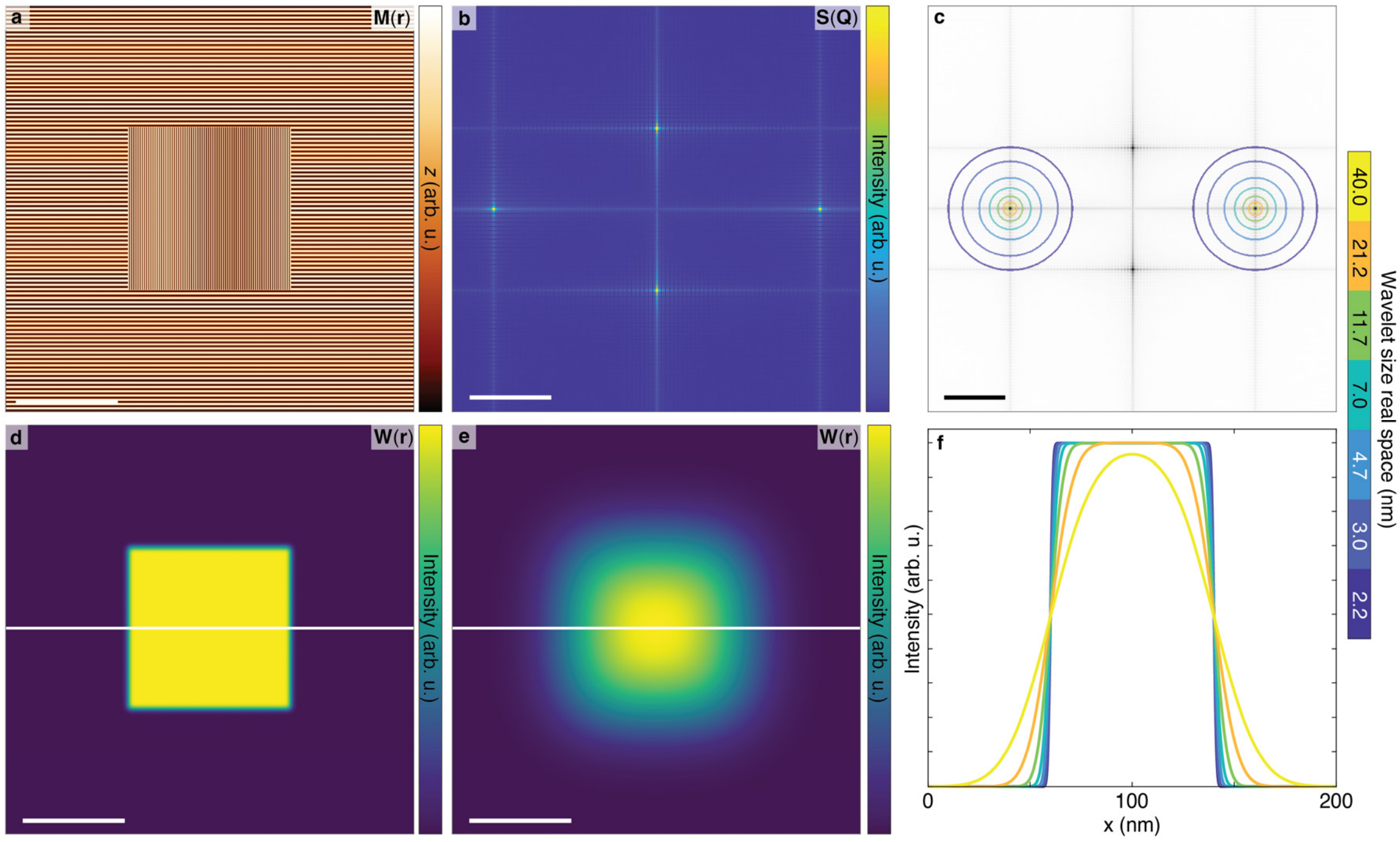

Fig. S6. Spatial resolution of the wavelet transformation. (a) Simulated magnetization image with two single-Q states (sine waves), i.e. $Q_1 = 6$ nm^{-1} in the inner box and $Q_2 = 3$ nm^{-1} in the rest of the image. **(b)** Corresponding **S**(**Q**)-map exhibiting Q-spots at Q_1 and Q_2. **(c)** Defined wavelets of varying sizes with colored outlines containing 90% of the wavelet intensity in Fourier space (corresponding real space sizes given in colorbar). **(d-e)** Wavelet transforms performed with two different wavelet sizes: ~2.2 nm for **(d)** and ~40 nm **(e)** in real space. The boundary between the Q_1 and Q_2-regions is smeared out the larger the wavelet size, as can also be seen in the line profiles along the white line shown in **(f)** (scale bar **M**(**r**) and **W**(**r**): 50 nm, scale bar **S**(**Q**): 3 nm^{-1}).

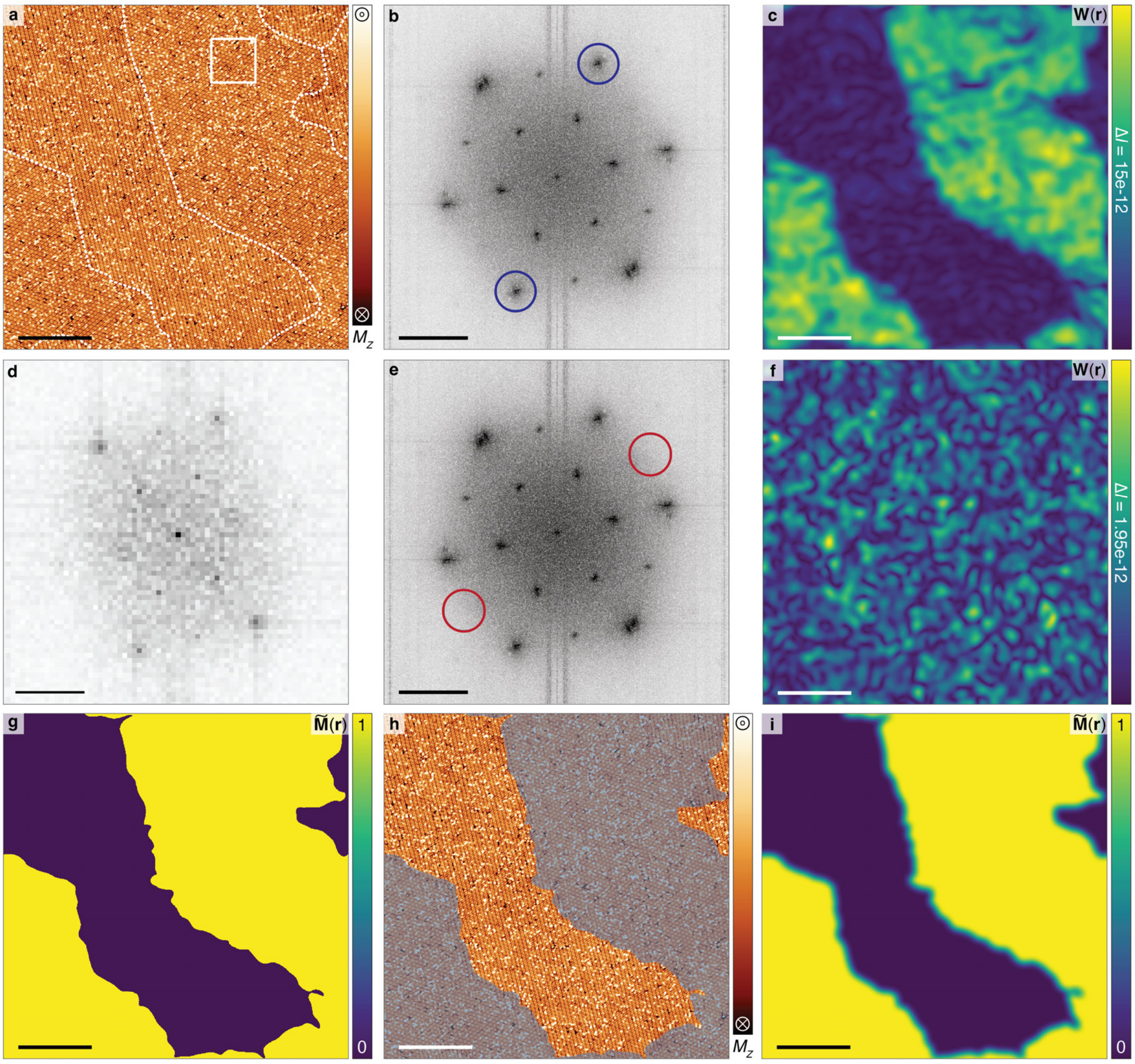

Fig. S7. Wavelet transformation method. (a) Magnetization image of the long-range ordered state at $T = 11$ K exhibits mainly two different domain types with domain walls drawn by eye (white dashed lines). **(b)** Corresponding **S**(**Q**) shows sharp *Q*-spots. One wavelet defined to address a single *Q*-spot overlayed over **S**(**Q**), where blue circles contain 90% of the wavelet intensity in *Q*-space. **(c)** Wavelet transform **W**(**r**) of **M**(**r**) shows intensity in one of the domains. **(d)** FFT of the small box indicated in **(a)** confirms the presence of the specific *Q*-state investigated in **(b, c)**. **(e)** Wavelet defined in **(c)** rotated away from the high symmetry axis to sample noise (red circles) overlayed over **S**(**Q**). **(f)** Wavelet transform **W**(**r**) of ***M***(***r***) in this background configuration shows a random distribution of intensity, which defines a background threshold. **(g)** Filtered wavelet image $\widetilde{\mathbf{M}}(\mathbf{r})$ of **(c)** after applying the background threshold to obtain the *Q*-area associated with the probed *Q*-spot. **(h)** Identified *Q*-area overlayed over magnetization image. **(i)** Wavelet image $\widetilde{\mathbf{M}}(\mathbf{r})$ after taking the uncertainty on the detected boundaries of the identified *Q*-area related to the finite wavelet size into account (image parameters: $I_t = 100$ pA; scale bar **M**(**r**), **W**(**r**) and $\widetilde{\mathbf{M}}(\mathbf{r})$: 50 nm, scale bar **S**(**Q**)-maps: 3 nm^{-1}).

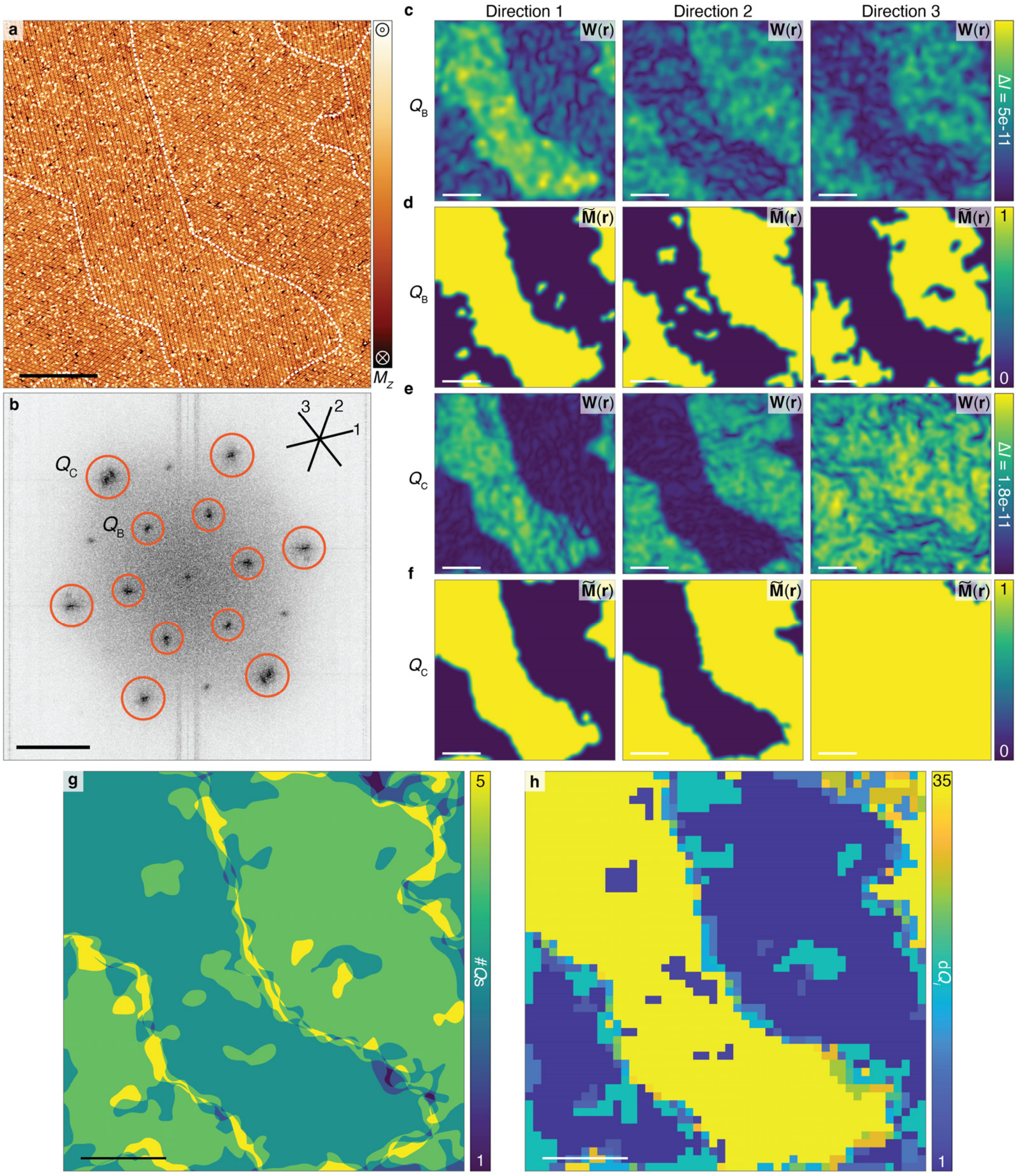

Fig. S8. Calibration of the wavelet method on the long-range ordered state. (a) Magnetization image $\mathbf{M}(\mathbf{r})$ in the long-range ordered state at $T = 11$ K exhibits mainly two different domain types with domain walls drawn by eye (white dashed lines). **(b)** Corresponding $\mathbf{S}(\mathbf{Q})$-image overlayed with orange circles containing 90% of the wavelet intensity in Q-space of the different wavelets (for each of the three high symmetry axes 1, 2, 3: one wavelet for Q_B and one for Q_C). **(c, e)** Resulting wavelet transformations $\mathbf{W}(\mathbf{r})$ for the different high symmetry axes: **(c)** Q_B and **(e)** Q_C. **(d, f)** Wavelet images $\tilde{\mathbf{M}}(\mathbf{r})$ after thresholding $\mathbf{W}(\mathbf{r})$ illustrate the spatial distributions of **(d)** Q_B- and **(f)** Q_C-periodicities approximately matching the domains in **(a)**. **(g)** Compilation of all $\tilde{\mathbf{M}}(\mathbf{r})$ (neglecting the uncertainty on the boundary) shows the multi-Q character of $\mathbf{M}(\mathbf{r})$. **(h)** Identified local Q-combinations dQ_i for a grid of boxes of size 4.7×4.7 nm^2, where each unique dQ_i is colored in a specific color. Two dominant domains (dark blue and yellow) are present (image parameters: $I_t = 100$ pA; scale bar $\mathbf{S}(\mathbf{Q})$: 3 nm^{-1}, scale bar other images: 50 nm).

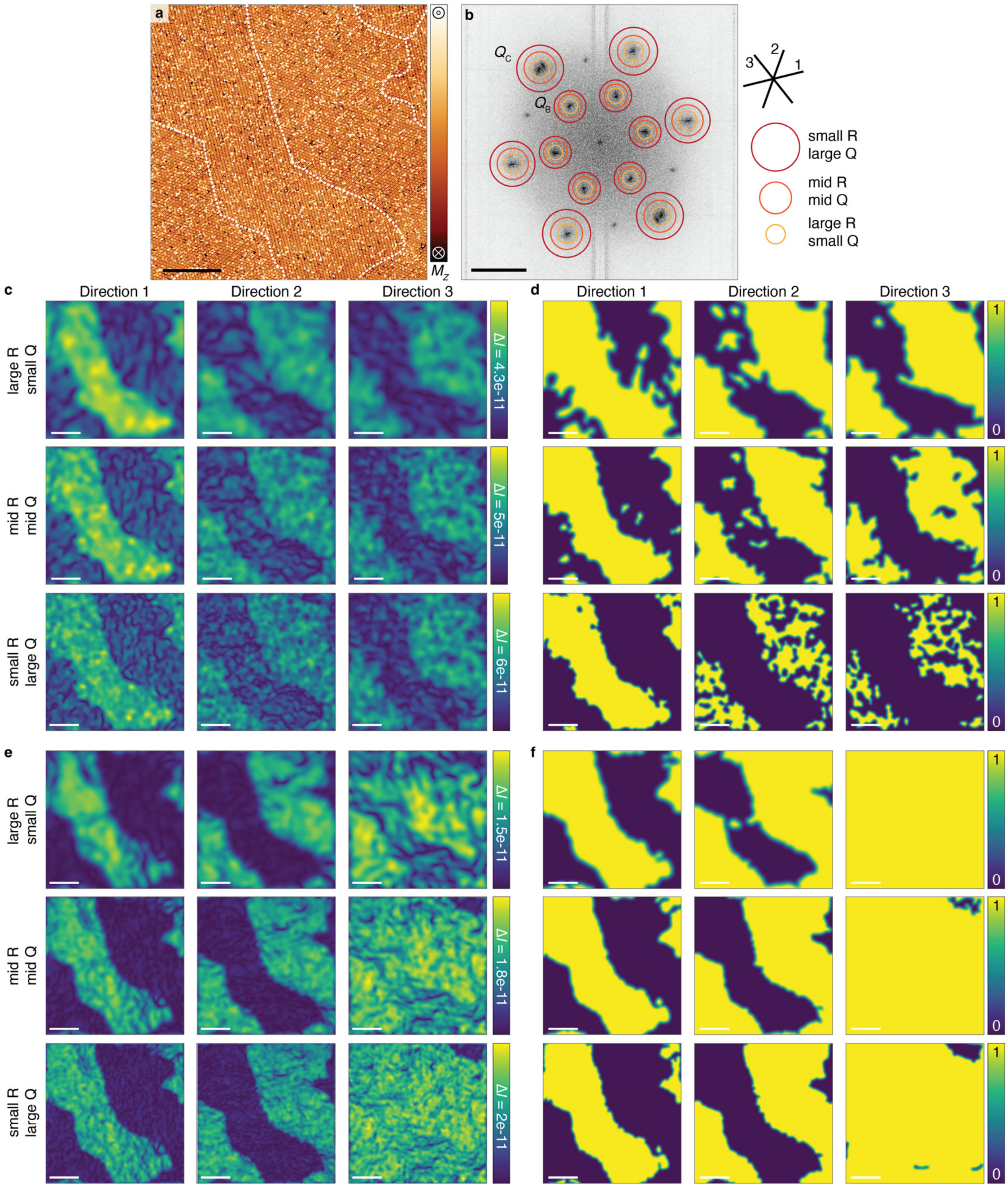

Fig. S9. Wavelet size dependence on wavelet transformation. (a) Magnetization image **M**(**r**) in the long-range ordered state at $T = 11$ K exhibits mainly two different domain types with domain walls drawn by eye (white dashed lines). **(b)** Corresponding **S**(**Q**) image overlayed with the contours of three different wavelet sizes containing 90% of the wavelet intensity in Q-space (R – real space, Q – Q-space). **(c, e)** Resulting wavelet transformations **W**(**r**) for the different high symmetry axes and wavelet sizes: **(c)** for Q_B and **(e)** for Q_C. **(d, f)** Corresponding wavelet images $\widetilde{\mathbf{M}}(\mathbf{r})$ illustrate the spatial distributions of **(d)** Q_B- and **(f)** Q_C-periodicities (image parameters: $I_t = 100$ pA; scale bar **M**(**r**), **W**(**r**) and $\widetilde{\mathbf{M}}(\mathbf{r})$: 50 nm, scale bar **S**(**Q**)-maps: 3 nm^{-1}).

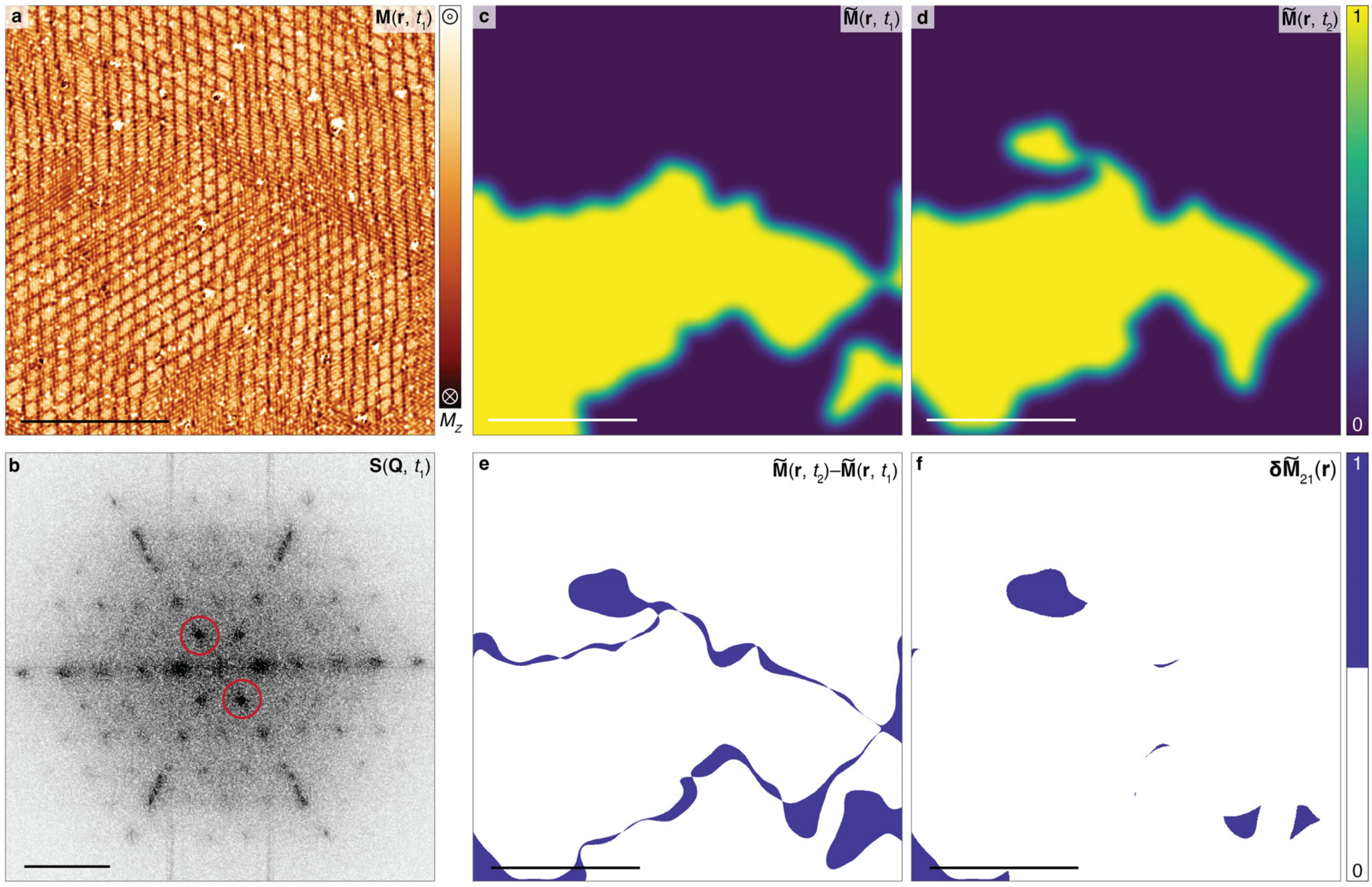

Fig. S10. Method to quantify *Q*-resolved changes in real space. (a) Real-space magnetization image $\mathbf{M}(\mathbf{r}, t_1)$ of Fig. 2 and **(b)** corresponding $\mathbf{S}(\mathbf{Q}, t_1)$ along with one exemplary wavelet as defined in Fig. 3. **(c-d)** Wavelet images $\widetilde{\mathbf{M}}(\mathbf{r}, t_1)$ and $\widetilde{\mathbf{M}}(\mathbf{r}, t_2)$ before and after the second field sweep to $B = 2$ T illustrating the evolution of the chosen Q_A-pocket in real space. **(e)** Subtraction of the wavelet images of **(c-d)** shows the areas where the Q_A-pocket either appeared or disappeared. **(f)** Spatial map of changed regions $\delta\widetilde{\mathbf{M}}_{21}(\boldsymbol{r})$ associated with fast dynamics related to the specific Q_A-pocket considering only changes outside of the uncertainty of $\widetilde{\mathbf{M}}(\boldsymbol{r}, t_1)$ (image parameters: $I_t = 200$ pA; scale bar $\mathbf{M}(\mathbf{r})$ and $\widetilde{\mathbf{M}}(\mathbf{r})$: 50 nm, scale bar $\mathbf{S}(\mathbf{Q})$-map: 3 nm^{-1}).

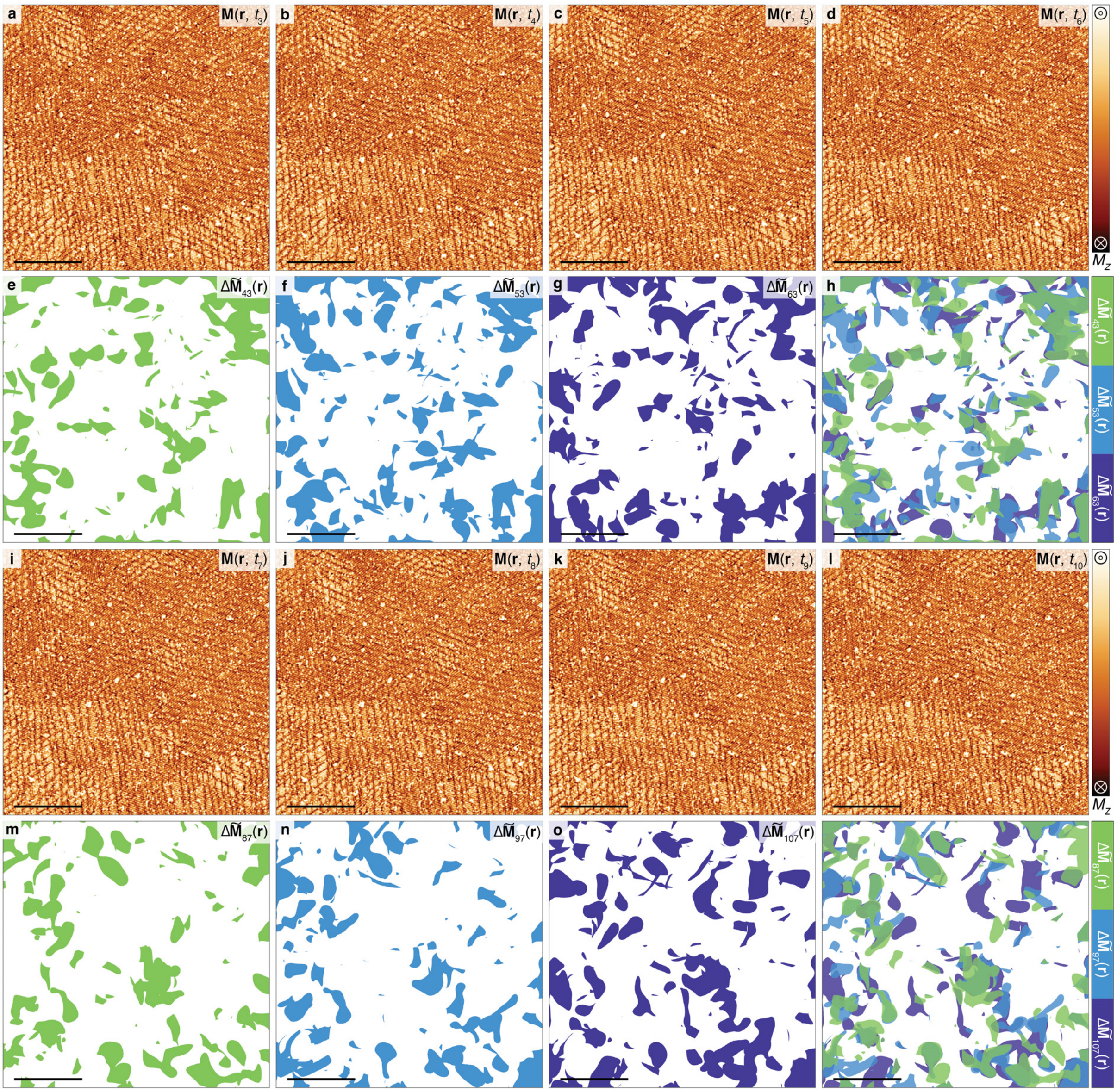

Fig. S11. Persistence of DH for further cycles. (a-d, i-l) Magnetization images $\mathbf{M}(\mathbf{r}, t_i)$ after the ith field cycle to $B = 2$T measured at $T = 1.3$ K and $B = 0$ T. **(e-g)** Wavelet filtered images $\Delta\widetilde{\mathbf{M}}_{i3}(\mathbf{r})$ with respect to the reference image at t_3. **(h)** Compilation of $\Delta\widetilde{\mathbf{M}}_{i3}(\mathbf{r})$ of **(e-g)** demonstrating the presence of DH. **(m-o)** Wavelet filtered images $\Delta\widetilde{\mathbf{M}}_{i7}(\mathbf{r})$ with respect to the reference image at t_7. **(p)** Compilation of $\Delta\widetilde{\mathbf{M}}_{i7}(\mathbf{r})$ of **(m-o)** revealing DH also for further cycles in the evolution of $\mathbf{M}(\mathbf{r}, t)$ (image parameters: $I_t = 200$ pA; scale bar $\mathbf{M}(\mathbf{r})$ and $\Delta\widetilde{\mathbf{M}}_{ij}(\mathbf{r})$: 50 nm; defect density of 0.0074 ML).

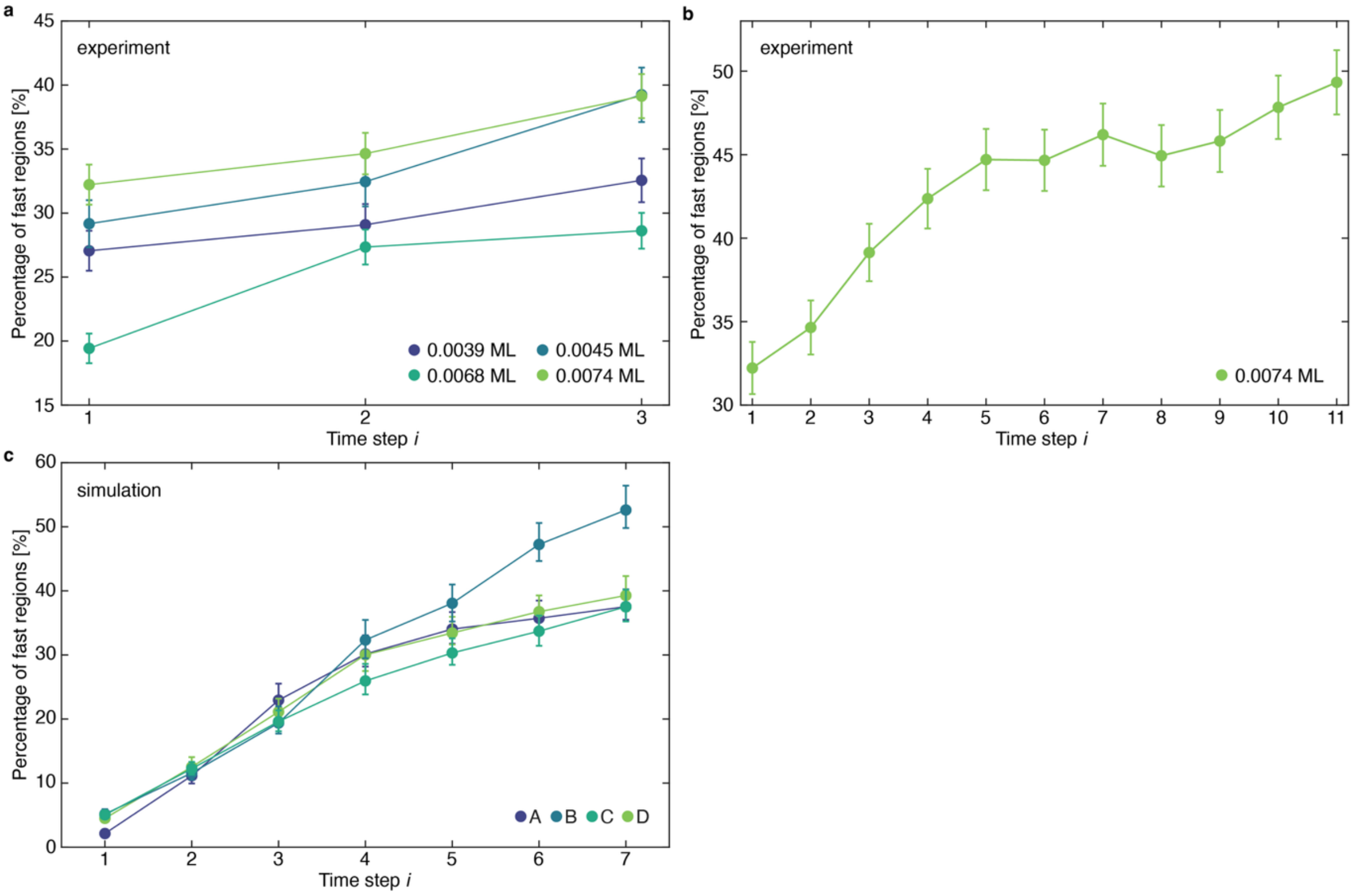

Fig. S12. Growth of fast regions over time. (a) Percentage of areas exhibiting fast dynamics compared to the total area for three consecutive time steps and for all the measured samples characterized by different defect densities (see Figs. 5d-f and S13) increases over time. **(b)** Percentage of areas exhibiting fast dynamics for one sample (see Fig. S11) for further field cycles corroborates the growth of fast regions. Error bars defined based on the uncertainty on the detected boundaries of the identified *Q*-areas related to the finite wavelet size. **(c)** Percentage of fast regions for the different ASD simulation runs (Figs. 8 and S20) also increases with time matching the experiment. Error bars were defined by varying the filtering threshold for the subtraction method by $\pm$ 8 %.

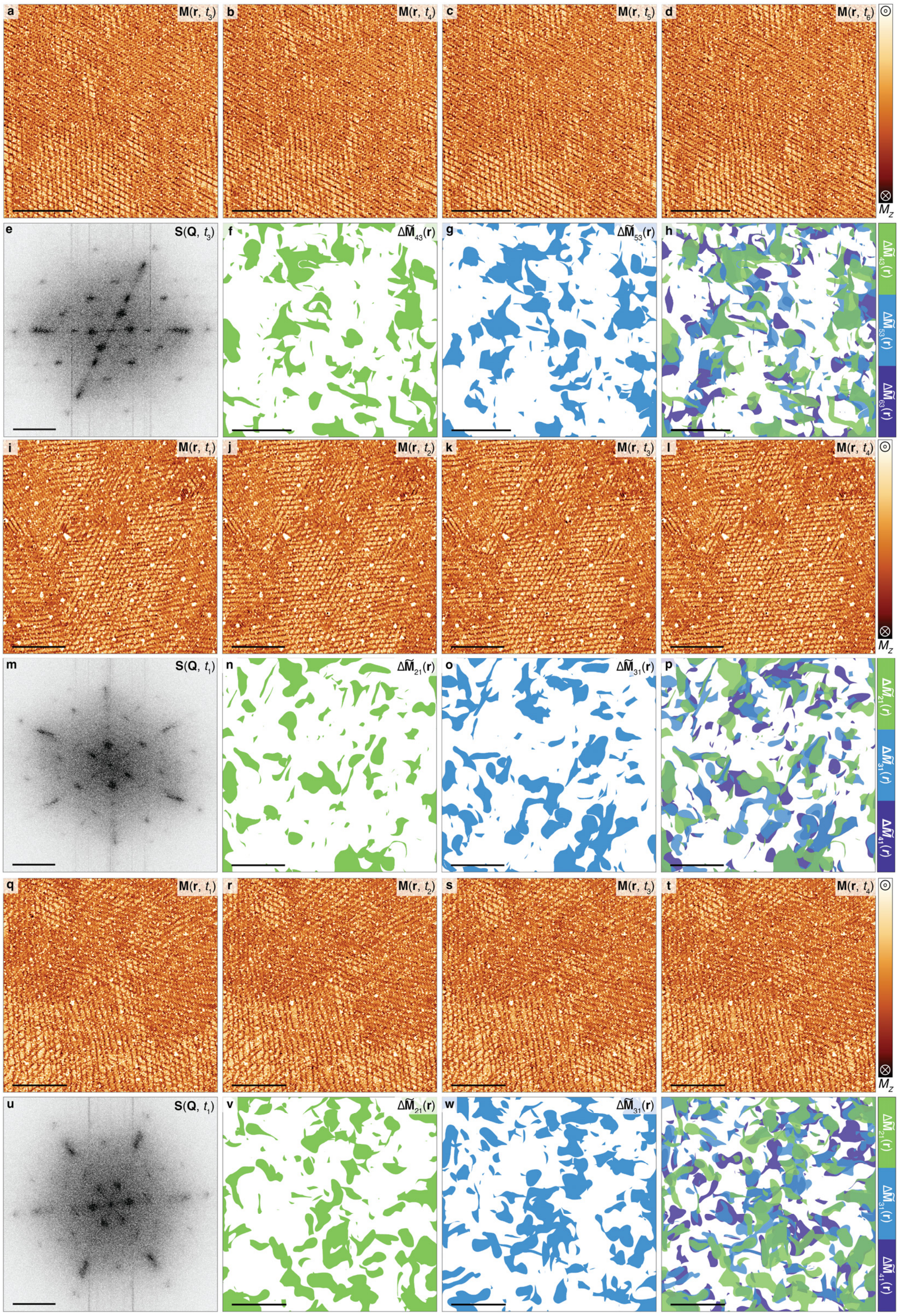

Fig. S13. Statistics of DH for three samples with varying defect densities. For each sample a series of magnetization images $\mathbf{M}(\mathbf{r}, t_i)$ after the ith field cycle to B = 2 T measured at T = 1.3 K and B = 0 T is shown along with an exemplary $\mathbf{S}(\mathbf{Q}, t_i)$. Additionally two wavelet images $\Delta\widetilde{\mathbf{M}}_{ij}(\mathbf{r})$ with respect to the reference image t_j and the compilation of them is shown in order to demonstrate the presence of DH: **(a-h)** sample with a defect density of 0.0039 ML, **(i-p)** sample with a defect density of 0.0068 ML and **(q-x)** sample with a defect density of 0.0074 ML (image parameters: I_t = 200 pA; scale bar $\mathbf{M}(\mathbf{r}, t_i)$ and $\Delta\widetilde{\mathbf{M}}_{ij}(\mathbf{r})$: 50 nm, scale bar $\mathbf{S}(\mathbf{Q}, t_i)$: 3 nm^{-1}).

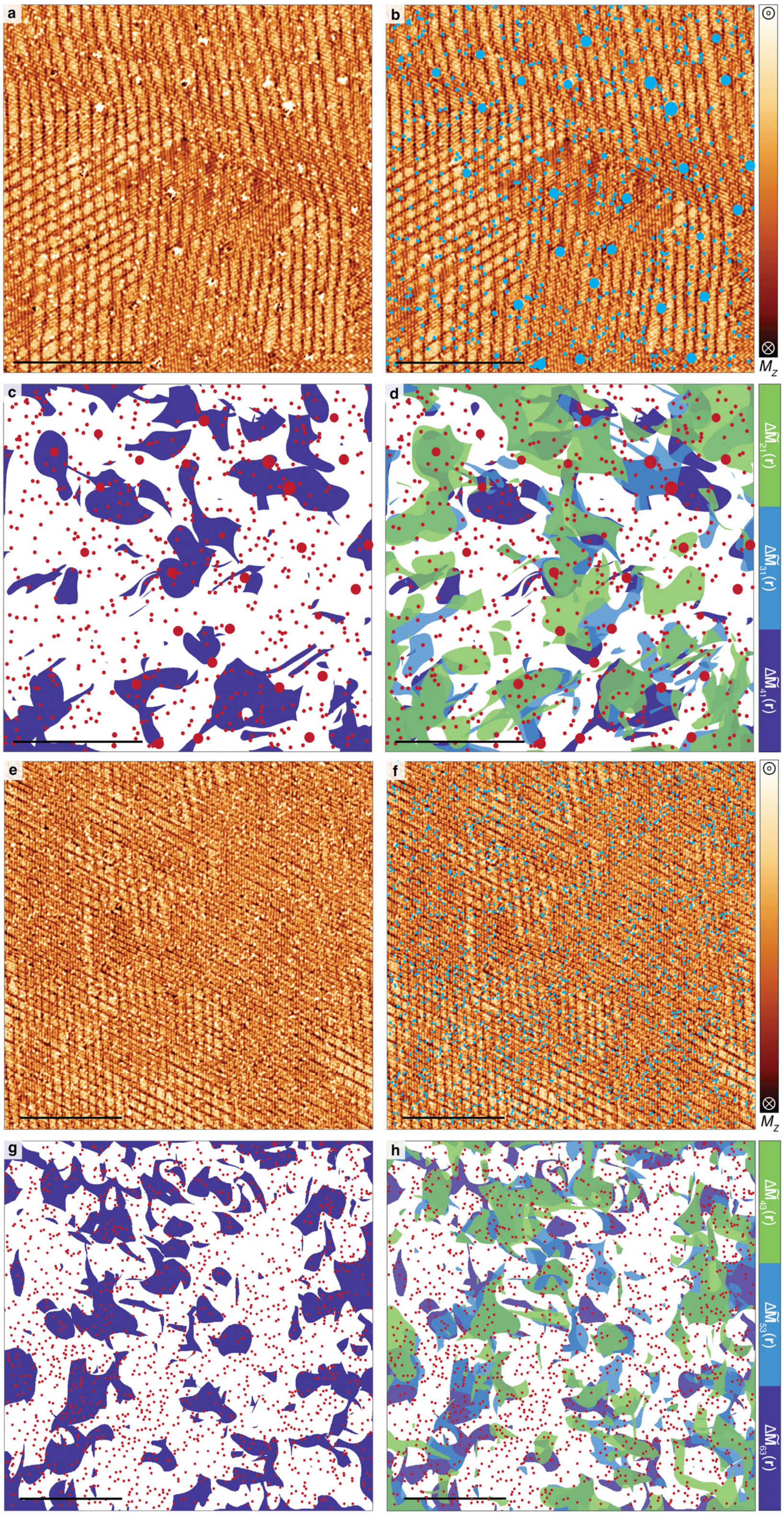

Fig. S14. Influence of defects on distribution of fast dynamics. (a) Spatially resolved magnetization image $\mathbf{M}(\mathbf{r}, t_1)$ of the sample with defect density 0.0045 ML used for Figs. 4 and 5. **(b)** $\mathbf{M}(\mathbf{r}, t_1)$ with defects indicated by blue dots. **(c)** Defect distribution identified in **(b)** (red dots) overlaid over the spatial map of fast dynamics $\Delta\widetilde{\mathbf{M}}_{41}(\mathbf{r})$. **(d)** Defect distribution (red dots) overlaid over the spatial map of fast dynamics for three consecutive time steps. **(e-h)** Same analysis repeated on a sample with defect density of 0.0039 ML using $\mathbf{M}(\mathbf{r}, t_2)$ to identify the defects (blue and red dots) (image parameters: $I_t = 200$ pA; scale bar: 50 nm).

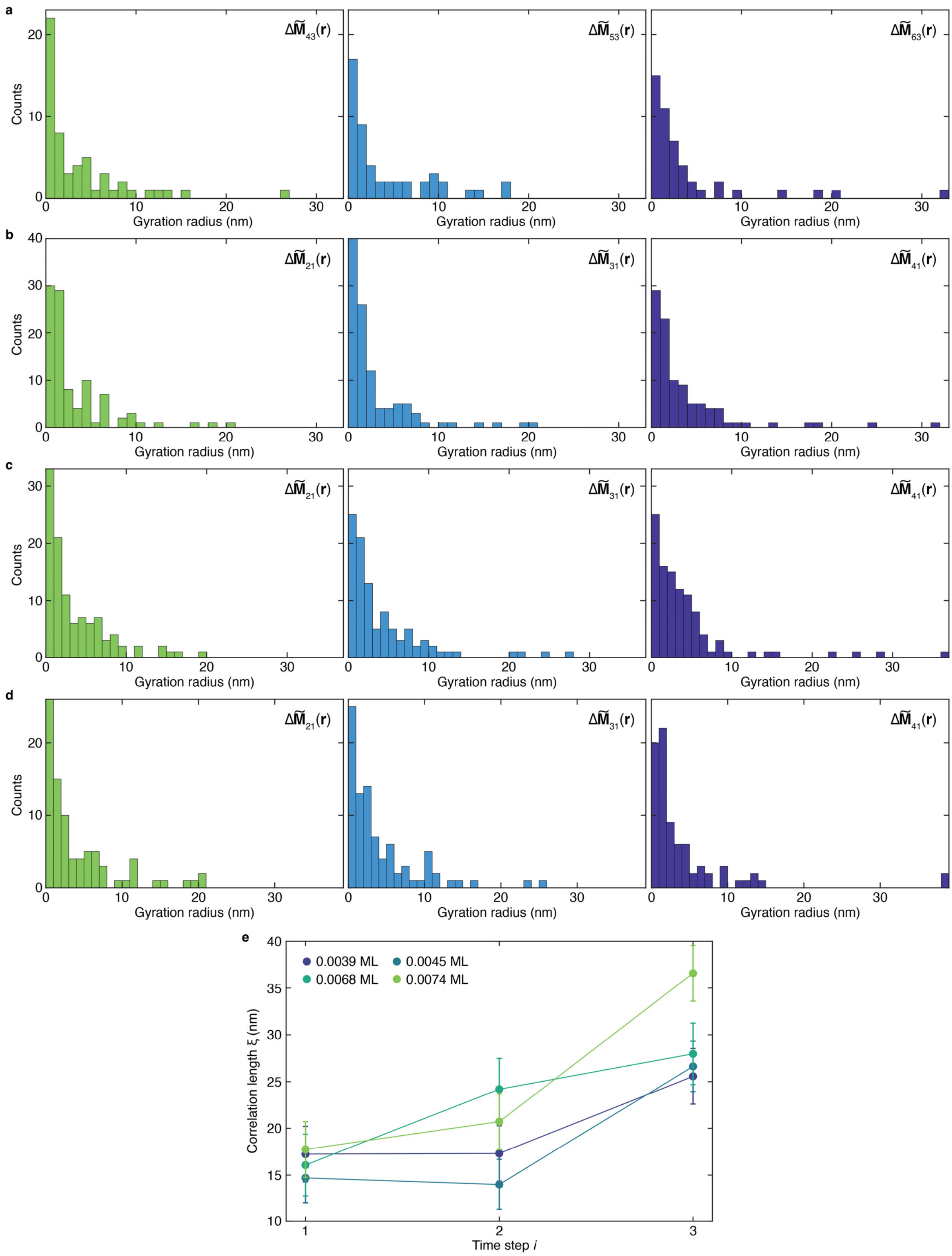

Fig. S15. Length scale of DH. (a-d) Histograms of the gyration radii R_S of the clusters of fast dynamics for three consecutive time steps for the different measured samples with defect densities of **(a)** 0.0039 ML, **(b)** 0.0045 ML, **(c)** 0.0068 ML and **(d)** 0.0074 ML. The analyzed $\Delta\widetilde{\mathbf{M}}_{ij}(\mathbf{r})$ images are shown in Figs. 5 and S13. **(e)** Corresponding correlation length ξ for the different samples defined as the average distance between two points within a cluster of fast dynamics increases over time. Error bars defined based on the uncertainty on the detected boundaries of the identified Q-areas related to the finite wavelet size.

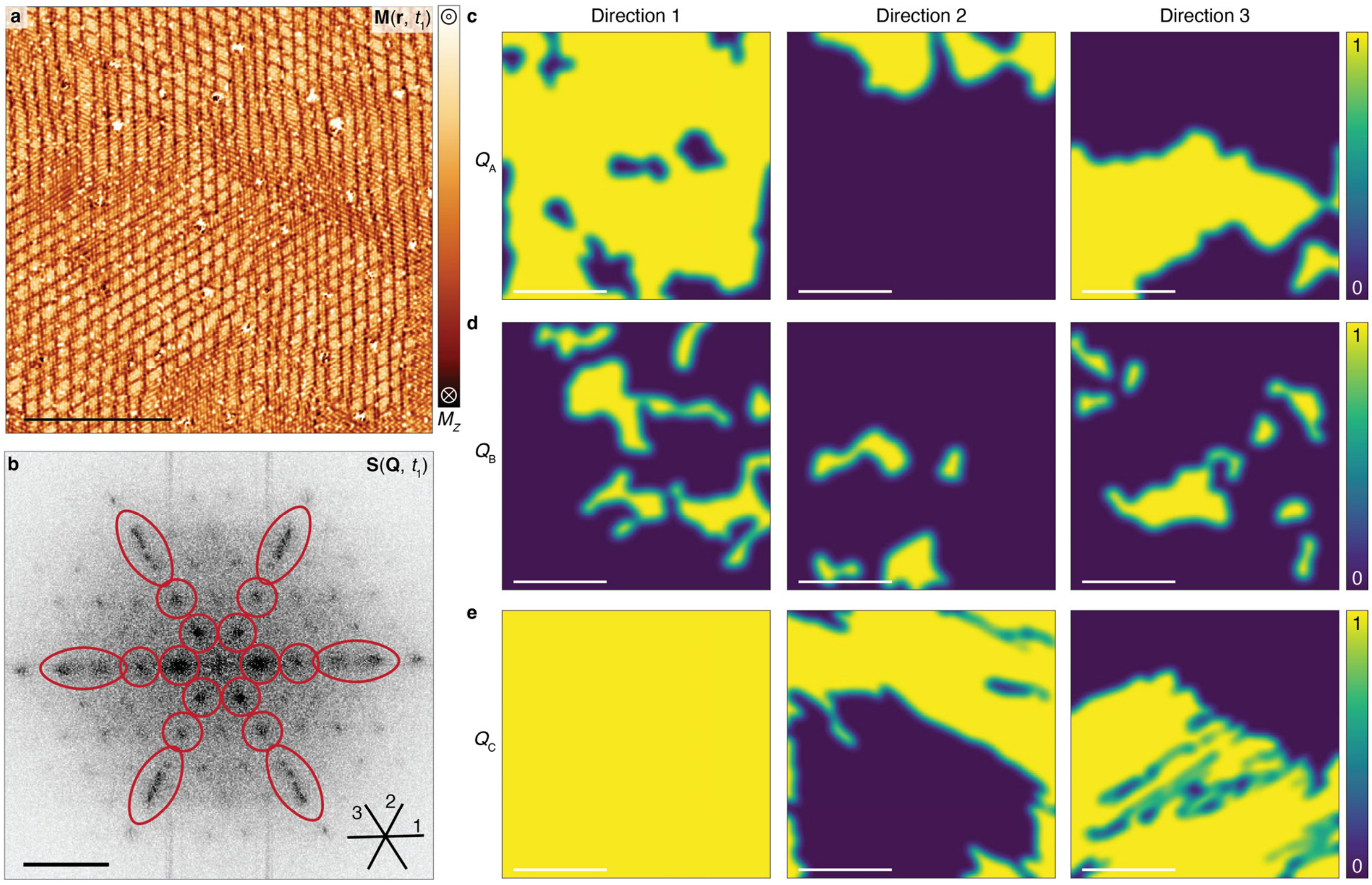

Fig. S16. *Q*-resolved wavelet analysis in the self-induced spin glass state. (a) Magnetization image **M**(**r**, t_1) of the sample with defect density 0.0045 ML and **(b)** corresponding **S**(**Q**, t_1) with red contours containing 90% of the wavelet intensity in *Q*-space for each wavelet used to sample the different *Q*-pockets. **(c-e)** Wavelet images $\tilde{\mathbf{M}}(\mathbf{r}, t_1)$ for the three high symmetry directions and for **(c)** the Q_A-pockets, **(d)** the Q_B-pockets and **(e)** the Q_C-pockets (image parameters: $I_t = 200$ pA; scale bar **M**(**r**, t_1) and $\tilde{\mathbf{M}}(\mathbf{r}, t_1)$: 50 nm, scale bar **S**(**Q**, t_1): 3 nm^{-1}, color scale for **S**(**Q**, t_1)-image is a linear gradient).

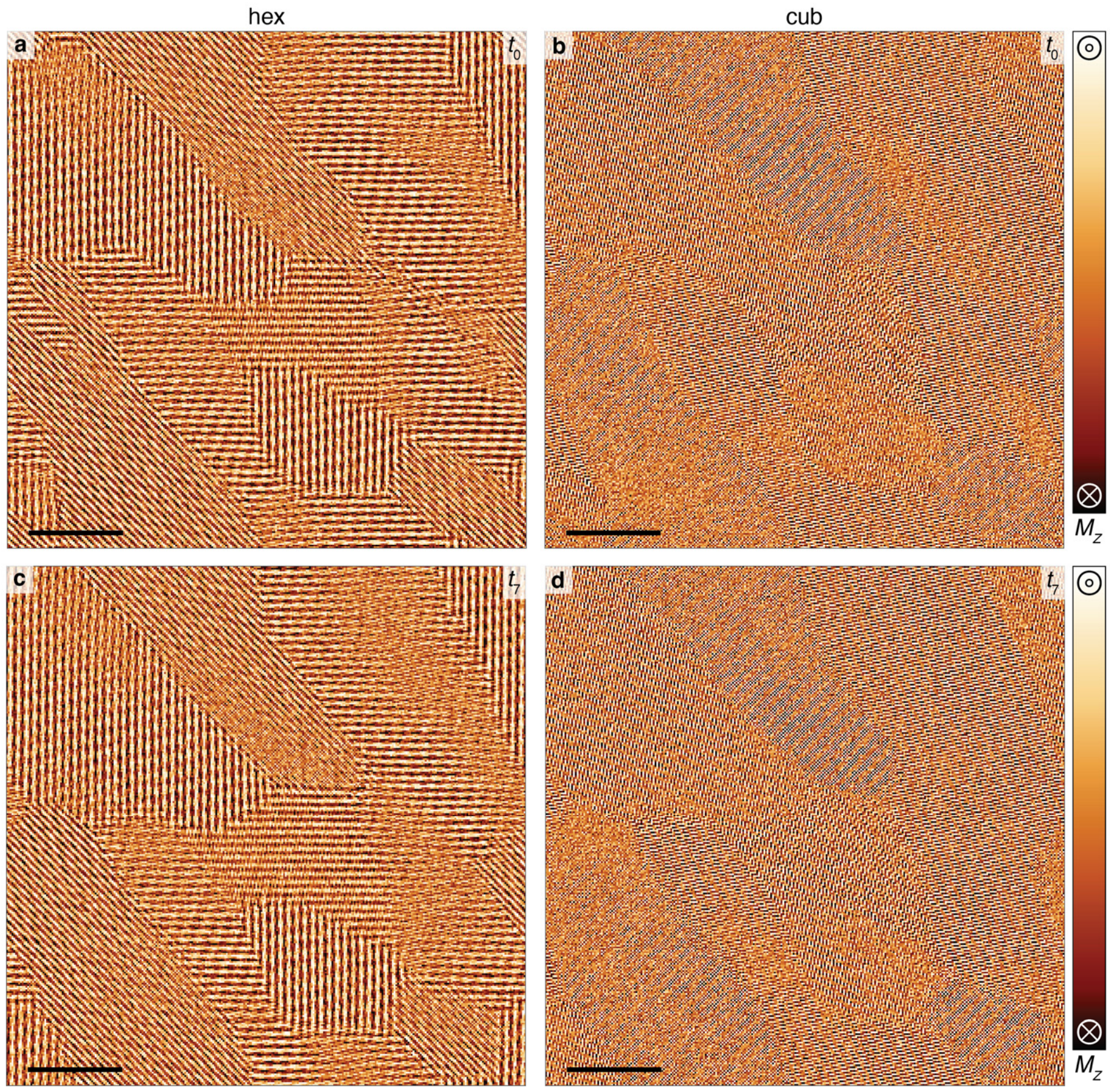

Fig. S17. Magnetization decomposed into the distinct sublattices. (a-b) Magnetization images of the top **(a)** hexagonal layer and **(b)** cubic layer at the beginning of the simulation (time t_0) illustrate the varying periodicities present. **(c-d)** Magnetization images of the top **(c)** hexagonal layer and **(d)** cubic layer at the end of the simulated time t_7 revealing a coexistence of stable and changing regions in both sublattices (scale bar: 20 nm).

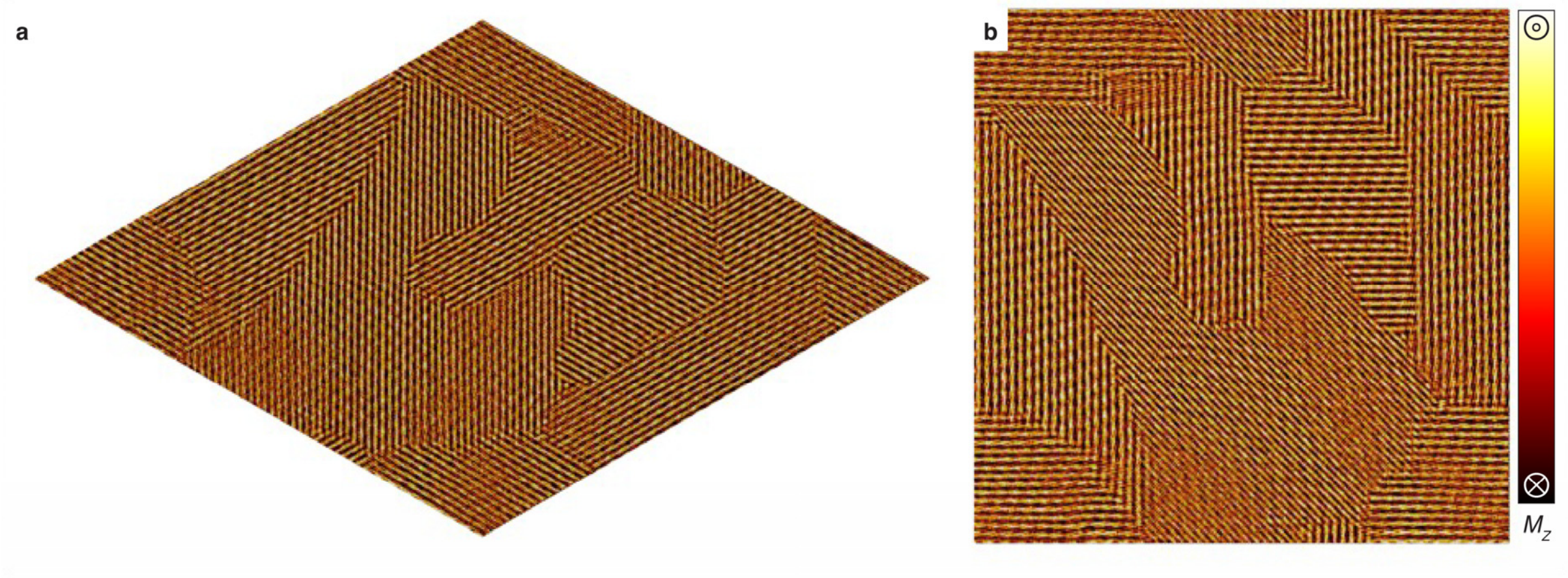

Fig. S18. Raw and skewed simulation data. (a) Undistorted texture of one atomic layer (hexagonal) in a 300 × 300 × 4 unit cell simulation box of dhcp Nd. **(b)** Magnetic texture of the same layer but projected onto a square image. Both images are represented as false-color images where the color indicates the out-of-plane component of the magnetization.

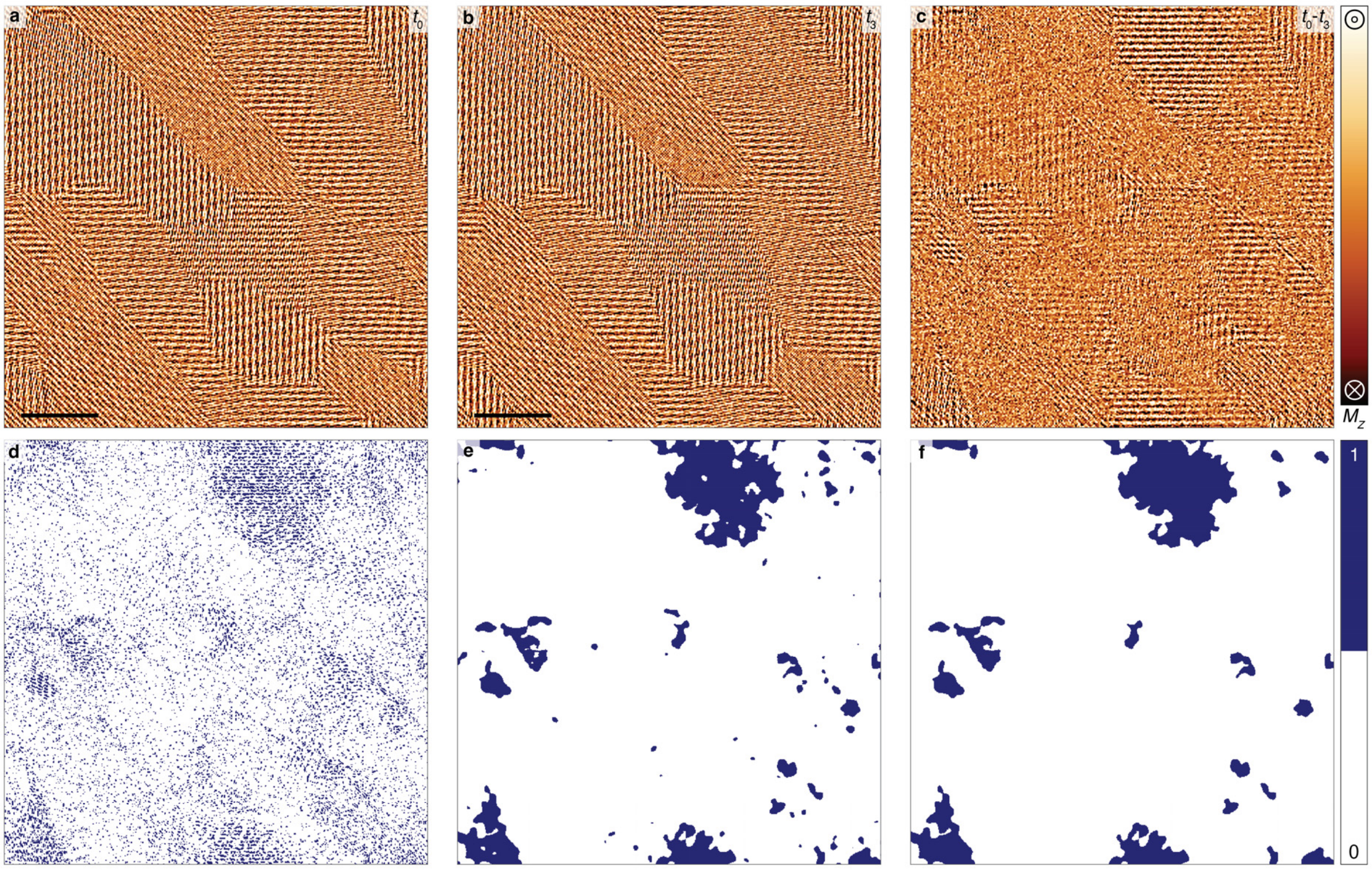

Fig. S19. Filtering method to quantify regions of change in real space. (a-b) Magnetization images at times t_0 and t_3. **(c)** Difference image of images shown in **(a)** and **(b)** exhibiting regions of strong as well as vanishing magnetic contrast. **(d)** Binary image representing positions of a different (blue) and stable (white) magnetization between t_0 and t_3 after extracting the positions of extremal contrast in **(c)**. **(e)** Identified connected regions of change in **(d)** based on a gaussian filter and additional threshold. **(f)** Finalized binary image representing stable (white) and changed (connected) regions (blue) between t_0 and t_3 after neglecting the smallest areas of change (scale bar: 20 nm).

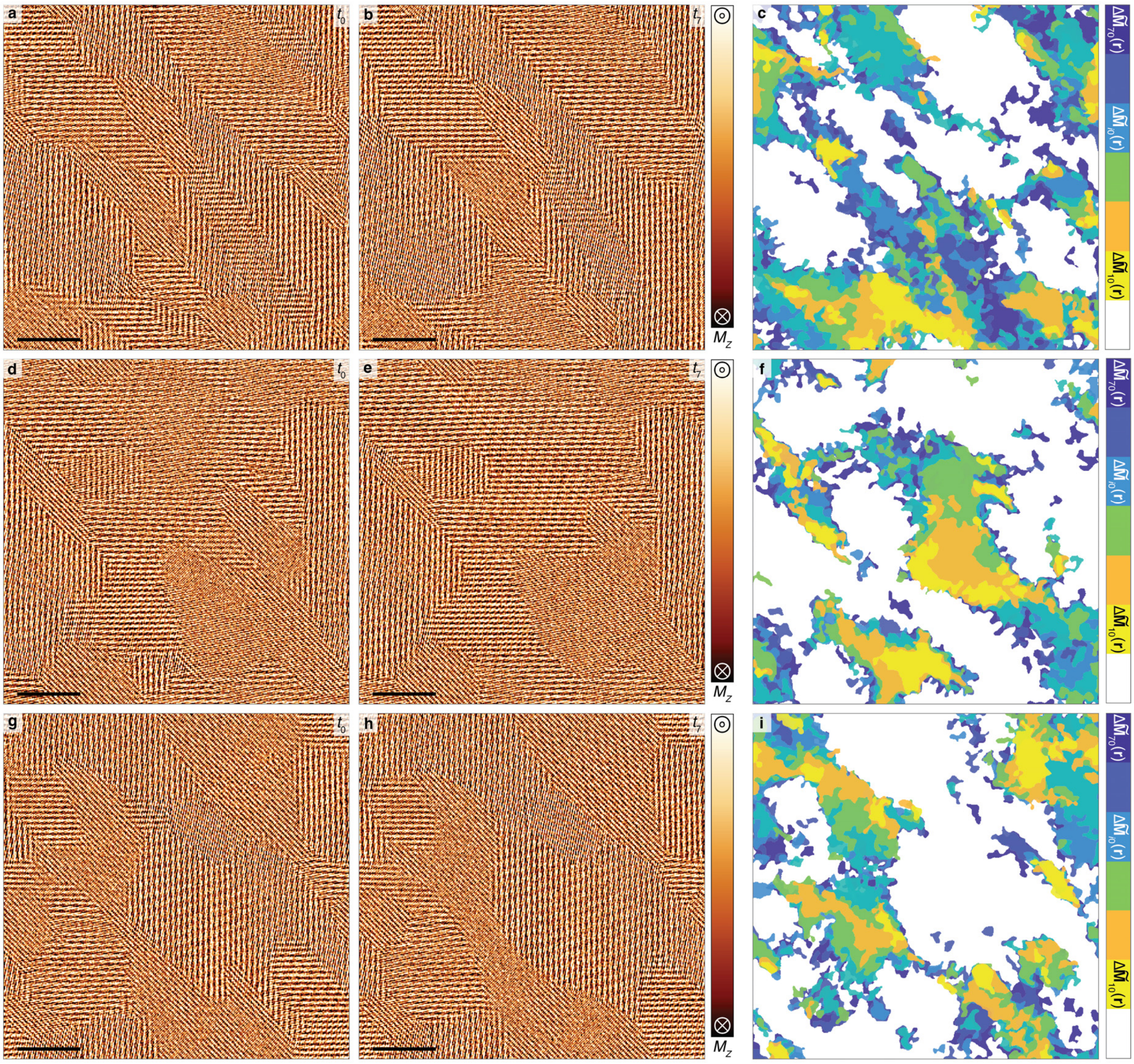

Fig. S20. DH for distinct ASD simulations. (a-b) Images showing snapshots of the magnetization at $t_0 = 1$ ps **(a)** and $t_7 = 500$ ps **(b)** for simulations of 300 × 300 × 4 unit cells of dhcp Nd. **(c)** Corresponding $\Delta\widetilde{\mathbf{M}}_{i0}(\mathbf{r})$ as a function of time (image at time t_i compared to initial state at t_0, $\Delta t = 70$ ps) indicate a coexistence of stable (white) and changing (colored) regions that nucleate at specific locations and then grow over time. **(d-f, g-i)** Same analysis for simulation runs with different starting states and heat-baths showing similar persistence behavior as well as a coexistence of stable and changing regions reminiscent of DH independent of the sample characteristics (scalebar: 20 nm).

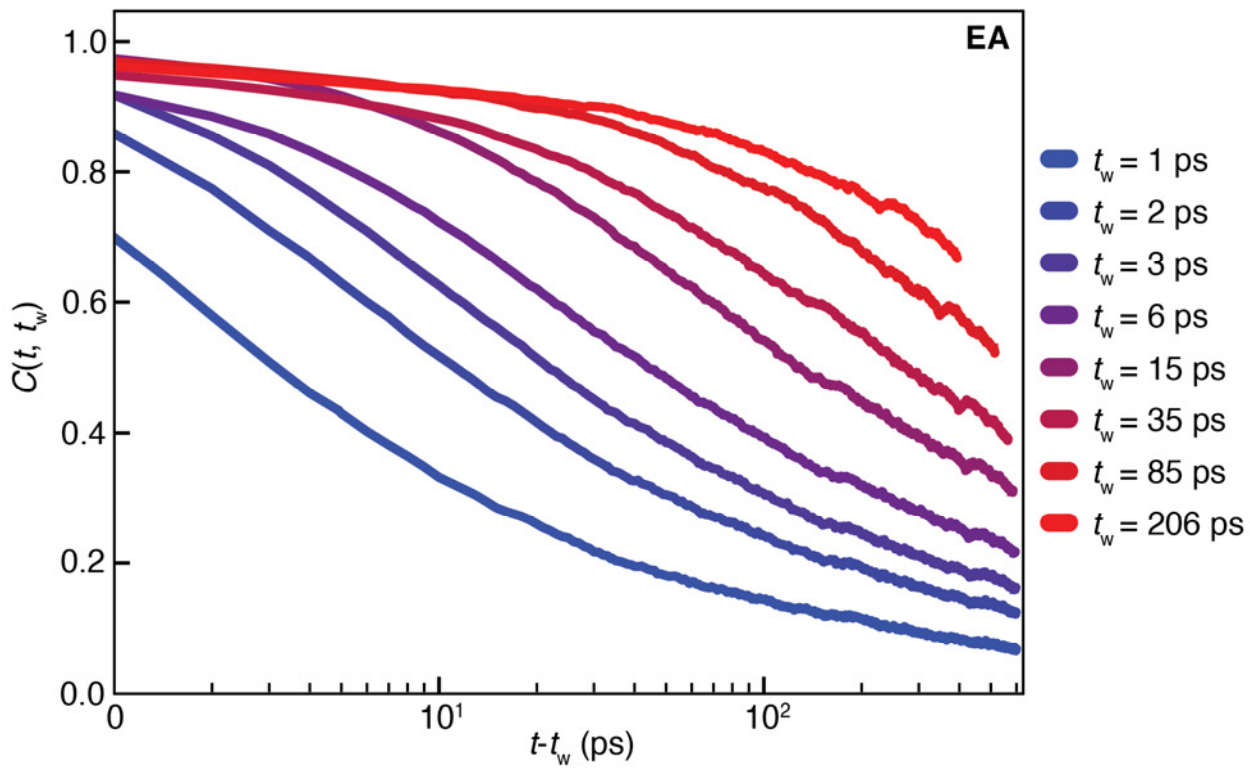

Fig. S21. Autocorrelation function. Simulated autocorrelation function $C(t, t_{\mathrm{w}})$ for a 300 × 300 × 4 unit cell simulation for an Edwards-Anderson (EA) model on the dhcp lattice (see Supplementary section S21 for a description of the methodology).
