# Supplementary material for: Dynamic heterogeneity in the self-induced spin glass state of elemental neodymium
Source: Nat Commun. 2026 Jun 20;17:7787. doi: 10.1038/s41467-026-74637-1 (PMC13439053; doi:10.1038/s41467-026-74637-1)
Supplement: Supplementary file 2 [file 41467_2026_74637_MOESM2_ESM.pdf]

nature portfolio

Peer Review File

Dynamic heterogeneity in the self-induced spin glass state of elemental neodymium

Corresponding Author: Professor Alexander Khajetoorians

This manuscript has been previously reviewed at another journal. This document only contains information relating to versions considered at Nature Communications.

This file contains all reviewer reports in order by version, followed by all author rebuttals in order by version.

Version 0:

Reviewer comments:

Reviewer #1

(Remarks to the Author)
The manuscript by Niggli et al. claims to demonstrate dynamical heterogeneity in the aging dynamics of Nd. They performed experiments using spin polarized STM to measure the local magnetization and also performed atomistic spin dynamics calculations.

The authors do a reasonably convincing job in demonstrating that there is spatial heterogeniety in the dynamics of the magnetization, showing that there are regions that remain frozen under magnetic field cycling. I am still a bit confused as to whether the results can be used to justify that the Nd is in a spin glass state. For instance, in a spin glass, one usually focuses on the dynamics of the two-time spin autocorrelation function, rather than the magnetization. It would be preferable if the authors could plot this, or at least indicate how their results relate to this two-time quantity.

Regarding aging dynamics, it is suggestive that the authors' spin dynamics calculations show dynamic heterogeneity that is qualitatively similar to that seen experimentally, and there does appear to be aging dynamics in the numerical simulations. However, to be more convincing, it would be helpful if there was more quantitative analysis of the dynamical heterogeneity. For instance, is there a lengthscale associated with the dynamical heterogeneity? Does this vary with time (or other parameters)? Can one determine the fractal dimension of the dynamic heterogeneities?

In order to meet the standards of Nature Communications, I would recommend that the authors work to add quantitative analysis of dynamic heterogeneities.

I would also suggest that supplementary Figure 17 be moved to the main text, as it makes the case for aging dynamics in a very succinct way.

Reviewer #2

(Remarks to the Author)
The manuscript ‘Dynamic heterogeneity in the self-induced spin glass state of elemental neodymium’ by Niggli et al. reports the experimental observation of magnetization changes on different time scales for different locations on a thick Nd film, accompanied by atomistic spin dynamics simulations. This work follows two previous papers by many of the same authors: Kamber et al., Science 2020, reported the measurement of multi-q / spin-glass behavior of Nd in real space, with some field-dependent measurements and a qualitative presentation of the aging; Verlhac et al., Nature Physics 2022, discussed the temperature dependence of the magnetic phases of Nd.

In the current work the emphasis is put on the quantitative evaluation of aging, specifically the identification of different

regions showing a change or no change in the magnetization pattern after a perturbation by a magnetic field pulse. The authors clearly demonstrate this effect and discuss in detail the concept of dynamic heterogeneity. In order to reveal and quantify the spatially changing time scales of magnetization dynamics an advanced analysis method was used. A mere image substraction method, as used in Fig. 2, does not yield very accessible results, which is somewhat surprising because the same method seems to work very well for the extraction of the spatial magnetization pattern as done in Extended Data Fig. 1. To obtain a better representation of local magnetization changes the advanced wavelet analysis method was employed, leading to a clearer picture which regions have changed due to the induced perturbations (Fig. 3), demonstrating the coexistence of regions with fast and slow magnetization changes. To my understanding this method uses some form of inverse Fourier transform which can correlate the contribution of a reciprocal wave vector with the spatial coordinates of the measurements, which is a common analysis method to localize the presence of specific periodicities in real space.

For this wavelet analysis all Q-pockets were used simultaneously. In principle this data also contains information about the dependence of the magnetization changes on the individual Q-vectors (length and possibly direction), which could potentially reveal which Q-vectors are more susceptible to aging. An analysis for separate Q-vectors (direction and length) was done for the long-range ordered state at T = 11 K, as presented in Supplementary Figs. S5, S6, S7. However, it is unclear to me if this analysis was also done for the low temperature phase, which is at the heart of this study. It would be interesting to know if such an analysis, where the Q-vectors/pockets were treated individually, did not yield any results, or if this deeper analysis of the data was not yet attempted. I see parts of this analysis in Supplementary Fig. S8, where the Q_A-pocket was analyzed separately. However, to my understanding the binary data in Fig. S8e should be a subset of the the data presented in Fig. 3d, which doesn’t seem to be the case. Therefore it is unclear to me what the main conclusion of such an analysis of individual Q-pockets is. Parts of this kind of analysis seem to be contained in an arxiv paper with the same title by the same authors. Unfortunately, and apparently due to previous lengthy review rounds, these interesting aspects were removed from the manuscript.

Overall I consider the measurements and the analysis to be of very high quality, the conclusions comprehensible, and the topic adequate, while at the same time somewhat narrow. While in principle I support publication in Nature Communications, for the benefit of the reader I suggest to consider to widen the scope and incorporate more aspects, e.g. an alalysis of the individual Q-pockets, into the main text (using some of the material in the supplemental information and possibly the previous arxiv version).

Minor comment: I suggest the authors add the information on the height range for the color scale for Fig. 2a,b,c, to enable a quantitative comparison with the data presented in Fig. 2e,f.

Open Access This Peer Review File is licensed under a Creative Commons Attribution 4.0 International License, which permits use, sharing, adaptation, distribution and reproduction in any medium or format, as long as you give appropriate credit to the original author(s) and the source, provide a link to the Creative Commons license, and indicate if changes were made.
In cases where reviewers are anonymous, credit should be given to 'Anonymous Referee' and the source.
The images or other third party material in this Peer Review File are included in the article’s Creative Commons license, unless indicated otherwise in a credit line to the material. If material is not included in the article’s Creative Commons license and your intended use is not permitted by statutory regulation or exceeds the permitted use, you will need to obtain permission directly from the copyright holder.

To view a copy of this license, visit https://creativecommons.org/licenses/by/4.0/

In the reply below, we make concise point by point replies. At the end of each reviewer reply, we summarize all the comprehensive changes made to the manuscript to address both reviewer comments. This will be duplicated for both reviewers.

Reviewer #1 (Remarks to the Author):

The manuscript by Niggli et al. claims to demonstrate dynamical heterogeneity in the aging dynamics of Nd. They performed experiments using spin polarized STM to measure the local magnetization and also performed atomistic spin dynamics calculations.

The authors do a reasonably convincing job in demonstrating that there is spatial heterogeneity in the dynamics of the magnetization, showing that there are regions that remain frozen under magnetic field cycling. I am still a bit confused as to whether the results can be used to justify that the Nd is in a spin glass state. For instance, in a spin glass, one usually focuses on the dynamics of the two-time spin autocorrelation function, rather than the magnetization. It would be preferable if the authors could plot this, or at least indicate how their results relate to this two-time quantity.

Regarding aging dynamics, it is suggestive that the authors' spin dynamics calculations show dynamic heterogeneity that is qualitatively similar to that seen experimentally, and there does appear to be aging dynamics in the numerical simulations. However, to be more convincing, it would be helpful if there was more quantitative analysis of the dynamical heterogeneity. For instance, is there a lengthscale associated with the dynamical heterogeneity? Does this vary with time (or other parameters)? Can one determine the fractal dimension of the dynamic heterogeneities?

In order to meet the standards of Nature Communications, I would recommend that the authors work to add quantitative analysis of dynamic heterogeneities.

I would also suggest that supplementary Figure 17 be moved to the main text, as it makes the case for aging dynamics in a very succinct way.

We thank the reviewer for their comments. We believe in this revision we have fulfilled the requests of the reviewer to add more quantitative information to the manuscript. To reply to the multiple inquiries mentioned:

- We agree that the two-time correlation function is one way to demonstrate aging dynamics. We have previously published the two-time correlation function of Nd(0001), twice (see refs. (*1, 2*)), and its comparison to standard spin models (e.g. EA model). We added the calculation in the supplement S17 to the main paper, as suggested. To avoid confusion, we have also shown in model calculations that a self-induced spin glass with a rugged energy landscape may have a more complex two-time correlation function (see ref. (*3*)). In general, this may require more information, such as done here, to demonstrate aging.
- It is not possible experimentally to relate our measurements of the magnetization to the two-time correlation function, as the reviewer suggests, as we do not measure dynamics with atomic resolution. We previously addressed that this is experimentally not possible, to date. We believe we have sufficiently demonstrated aging: (1) in a previous manuscript (ref. (*1*)) by showing the presence of multiple relaxation times (i.e. time scales), which indirectly relates to the two-time correlation function, and (2)

as shown in this revision, by showing that neodymium exhibits continuously evolving local order, which depends on the previous state, and never relaxes to a fixed state after subsequent perturbations. Nearly all experiments in literature concern bulk ensemble measurements, where a thermodynamic quantity (e.g. susceptibility) is measured over time in response to a perturbation. This does include measurements of the magnetization or magnetic susceptibility (*4*), and is also used to infer the two-time correlation function. None of these measurements captures information about the local order, which is precisely the new aspect of our method.

- As suggested, we have now added two quantitative analysis methods of dynamic heterogeneity and a detailed discussion to the manuscript.
    1. We calculated the percentage of regions that exhibit fast dynamics and compared this to the total region for both the experiment as well as the simulations. In both cases, we observe an increase of this percentage over time, as shown in Extended Data Figure 4, quantifying the nucleation behavior.
    2. Second, we performed a new analysis to provide a crude estimate of the length scales that emerged. We quantitatively analyzed the irregularly shaped connected clusters of fast dynamics in $\Delta\widetilde{\boldsymbol{M}}_{ij}(\boldsymbol{r})$-images by calculating a so-called gyration radius R_s and a correlation length ξ based on it, following refs. (*5, 6*). R_s denotes the average distance from a point in a specific connected cluster of fast dynamics of size s in the $\Delta\widetilde{\boldsymbol{M}}_{ij}(\boldsymbol{r})$-images to its center of mass, whereas ξ is a measure of the average distance between two points within a connected region of fast dynamics. The calculated correlation length increases from ~15-18 nm after the first perturbation to ~25-35 nm after the third. However, DH in neodymium is not fully described by such a single length scale, as the distributions of gyration radii for the various clusters of fast dynamics present in a given area are broad. Overall, there exist many clusters of fast dynamics with gyration radii below 10 nm along with a small number of clusters exhibiting radii in the range of ~15-38 nm (see S11 for details). These broad distributions of R_s demonstrate the complexity of the heterogeneous dynamics observed in neodymium, likely connected to its multi-*Q* nature.

Comprehensive changes to the manuscript:

1. Added the two-time autocorrelation function of Fig. S17a to the main text (new Fig. 1e) as asked and referred to previous examples.
2. Added a figure and a description about how magnetic cycling can be used to anneal/perturb the self-induced spin glass state.
3. Added discussion and new figure relating the experimental snapshots to phase space evolution and both history and aging.
4. Added a quantitative analysis of the length scale of DH, using a new method, and added a discussion to the paper.
5. Added a figure and a discussion of the effect of defects on DH to the manuscript, and how we can differentiate between defect pinning and DH.
6. Added *Q*-decomposition figures and explanation of how the local system is multi-*Q* and therefore one cannot use single Q's as a delineation to learn about the dynamics.

7. Explained the difference between wavelets and inverse Fourier transforms and added a short introduction to the wavelet concept.
8. Added a height comparison to new Fig. 4 as recommended by reviewer 2.
9. Fixed the mistake in Fig. S8f (new Fig. S7f) brought up by reviewer 2.

Reviewer #2 (Remarks to the Author):

The manuscript 'Dynamic heterogeneity in the self-induced spin glass state of elemental neodymium' by Niggli et al. reports the experimental observation of magnetization changes on different time scales for different locations on a thick Nd film, accompanied by atomistic spin dynamics simulations. This work follows two previous papers by many of the same authors: Kamber et al., Science 2020, reported the measurement of multi-q / spin-glass behavior of Nd in real space, with some field-dependent measurements and a qualitative presentation of the aging; Verlhac et al., Nature Physics 2022, discussed the temperature dependence of the magnetic phases of Nd.

In the current work the emphasis is put on the quantitative evaluation of aging, specifically the identification of different regions showing a change or no change in the magnetization pattern after a perturbation by a magnetic field pulse. The authors clearly demonstrate this effect and discuss in detail the concept of dynamic heterogeneity. In order to reveal and quantify the spatially changing time scales of magnetization dynamics an advanced analysis method was used. A mere image subtraction method, as used in Fig. 2, does not yield very accessible results, which is somewhat surprising because the same method seems to work very well for the extraction of the spatial magnetization pattern as done in Extended Data Fig. 1. To obtain a better representation of local magnetization changes the advanced wavelet analysis method was employed, leading to a clearer picture which regions have changed due to the induced perturbations (Fig. 3), demonstrating the coexistence of regions with fast and slow magnetization changes. To my understanding this method uses some form of inverse Fourier transform which can correlate the contribution of a reciprocal wave vector with the spatial coordinates of the measurements, which is a common analysis method to localize the presence of specific periodicities in real space.

For this wavelet analysis all Q-pockets were used simultaneously. In principle this data also contains information about the dependence of the magnetization changes on the individual Q-vectors (length and possibly direction), which could potentially reveal which Q-vectors are more susceptible to aging. An analysis for separate Q-vectors (direction and length) was done for the long-range ordered state at T = 11 K, as presented in Supplementary Figs. S5, S6, S7. However, it is unclear to me if this analysis was also done for the low temperature phase, which is at the heart of this study. It would be interesting to know if such an analysis, where the Q-vectors/pockets were treated individually, did not yield any results, or if this deeper analysis of the data was not yet attempted. I see parts of this analysis in Supplementary Fig. S8, where the Q_A-pocket was analyzed separately. However, to my understanding the binary data in Fig. S8e should be a subset of the the data presented in Fig. 3d, which doesn't seem to be the case. Therefore it is unclear to me what the main conclusion of such an analysis of individual Q-pockets is. Parts of this kind of analysis seem to be contained in an arxiv paper with the same title by the same authors. Unfortunately, and

apparently due to previous lengthy review rounds, these interesting aspects were removed from the manuscript.

Overall I consider the measurements and the analysis to be of very high quality, the conclusions comprehensible, and the topic adequate, while at the same time somewhat narrow. While in principle I support publication in Nature Communications, for the benefit of the reader I suggest to consider to widen the scope and incorporate more aspects, e.g. an alalysis of the individual Q-pockets, into the main text (using some of the material in the supplemental information and possibly the previous arxiv version).

Minor comment: I suggest the authors add the information on the height range for the color scale for Fig. 2a,b,c, to enable a quantitative comparison with the data presented in Fig. 2e,f.

- We thank the reviewer for the comments and positive feedback. Concerning the analysis: during the previous review process, we were asked to provide more quantitative methods besides image subtraction and area identification to quantify DH. Therefore, we adapted a rigorous wavelet analysis method in later drafts, which is not present in the arxiv version.
- Concerning the comment about subtraction: as shown in Extended Data Fig. 1, we extracted the magnetization from two images measured at two different bias voltages. However, these images are measured simultaneously through a multi-pass acquisition, i.e. each line of the image is scanned at both voltages. This means that the two images can be precisely subtracted to give $\boldsymbol{M}(\boldsymbol{r})$. In contrast, consecutively measured $\boldsymbol{M}(\boldsymbol{r}, t)$ images after magnetic field cycling are acquired hours apart and have to be aligned with each other using defects, as the application of a magnetic field can introduce small lateral drifts. Consequently, a simple image subtraction method is not sufficient to quantify differences in the local multi-Q order. Nevertheless, this was not deemed sufficiently deep analysis by previous reviewers. We believe subtraction does reveal qualitative features of DH, but that the wavelet analysis, or something similar, is necessary to be more quantitative.
- In order to avoid confusion, we should mention that wavelet analysis and inverse FFTs (iFFTs) are similar, but not the same thing. FFTs and iFFTs are loosely based on sines and cosines. One can filter an FFT and process it using an iFFT to recover Q-resolved real space information. However, this typically means truncating a sine wave, which can lead to frequency artifacts. A wavelet is based on a finite length wavepacket with a defined waveform, where the frequency and amplitude modulation is controlled. This has particular advantages for certain types of problems like trying to identify specific patterns where both real space and reciprocal space information is needed simultaneously. We have added this comment to the paper.
- We agree that doing a Q-resolved study of the real-space snapshots is extremely exciting. We found that a Q-resolved analysis is not necessarily straightforward to interpret the physics, due to the multi-Q nature. To illustrate this we performed a new qualitative study: we decomposed two different Q-pockets using the wavelet analysis for different snapshots as shown in Fig. 7a-h. The changes in the total area of each Q-pocket may be related to how its favorability changes in response to magnetic field cycling. However, this statement may only be made if the dynamics of a single Q would be independent of the other possible Q-values, i.e. when we have single-Q

patterns. This is not the case for neodymium, as multiple *Q*-pockets are often present in the same location, as can be seen in the example in Fig. 7i-l, where the spatial overlap is moreover time-dependent. This indicates that the dynamics of the different *Q* possibilities are coupled, i.e. that the local order is multi-*Q* like with multiple possible combinations of *Q*-states. Therefore, the decomposition to be made may not be single *Q*-specific, but rather pattern specific (i.e. each multi-*Q* pattern). We identified >30 patterns in the films studied, and we do not have sufficient statistics in the areas we studied to analyze the evolution of all the patterns in one snapshot. Therefore, the proper treatment of understanding the spatiotemporal evolution with a wavelet analysis of the self-induced spin glass state would require the identification of all the various patterns. To be explicit: DH does not involve a *Q*-dependence and such a study would go beyond the claim of seeing DH. We believe this is one of the exciting aspects to explore in the future, but it also requires more statistics. We added a passage and new figures to address this.

- We thank the reviewer for spotting a mistake in Fig. S8. It is Fig. S8f (the same regions as Fig S8e after taking into account the uncertainty on the detected boundaries) that is a subset of the fast dynamics shown in the main text (new Fig. 5d). We have updated the figure to show the correct image.

Comprehensive changes to the manuscript:

1. Added the two-time autocorrelation function of Fig. S17a to the main text (new Fig. 1e) as asked and referred to previous examples.
2. Added a figure and a description about how magnetic cycling can be used to anneal/perturb the self-induced spin glass state.
3. Added discussion and new figure relating the experimental snapshots to phase space evolution and both history and aging.
4. Added a quantitative analysis of the length scale of DH, using a new method, and added a discussion to the paper.
5. Added a figure and a discussion of the effect of defects on DH to the manuscript, and how we can differentiate between defect pinning and DH.
6. Added *Q*-decomposition figures and explanation of how the local system is multi-*Q* and therefore one cannot use single Q's as a delineation to learn about the dynamics.
7. Explained the difference between wavelets and inverse Fourier transforms and added a short introduction to the wavelet concept.
8. Added a height comparison to new Fig. 4 as recommended by reviewer 2.
9. Fixed the mistake in Fig. S8f (new Fig. S7f) brought up by reviewer 2.

References

1. U. Kamber *et al.*, Self-induced spin glass state in elemental and crystalline neodymium. *Science* **368**, eaay6757 (2020).
2. B. Verlhac *et al.*, Thermally induced magnetic order from glassiness in elemental neodymium. *Nat. Phys.* **18**, 905-911 (2022).

3. A. Kolmus, M. I. Kasnelson, A. A. Khajetoorians, H. J. Kappen, Atom-by-atom construction of attractors in a tunable finite size spin array. *New. J. Phys.* **22**, 023038 (2020).
4. E. Vincent, in *Ageing and the Glass Transition,* M. Henkel, M. Pleimling, R. Sanctuary, Eds. (Springer Berlin Heidelberg, Berlin, Heidelberg, 2007), pp. 7-60.
5. D. Stauffer, *Introduction to percolation theory*. A. Aharony, Ed., (CRC Press, ed. Rev. 2nd., 1994).
6. C. Frydendahl, Fractal analysis package (https://ch.mathworks.com/matlabcentral/fileexchange/71770-fractal-analysis-package). *MATLAB Central File Exchange*, (2026).
